# ABC inference of multi-population divergence with admixture from unphased population genomic data

**DOI:** 10.1111/mec.12881

**Published:** 2014-09-06

**Authors:** John D Robinson, Lynsey Bunnefeld, Jack Hearn, Graham N Stone, Michael J Hickerson

**Affiliations:** * Department of Biology, City College of New York160 Convent Ave., MR 526, New York, NY, 10031, USA; † Institute of Evolutionary Biology, University of Edinburgh, Ashworth LaboratoriesKings Buildings, West Mains Road, Edinburgh, EH9 3JT, UK; ‡ Subprogram in Ecology Evolution and Behavior, The Graduate Center of the City University of New YorkNew York, NY, 10016, USA; § Division of Invertebrate Zoology, American Museum of Natural HistoryNew York, NY, 10024, USA

**Keywords:** approximate Bayesian computation, *Biorhiza pallida*, gene flow, next-generation sequencing, phylogeography, speciation

## Abstract

Rapidly developing sequencing technologies and declining costs have made it possible to collect genome-scale data from population-level samples in nonmodel systems. Inferential tools for historical demography given these data sets are, at present, underdeveloped. In particular, approximate Bayesian computation (ABC) has yet to be widely embraced by researchers generating these data. Here, we demonstrate the promise of ABC for analysis of the large data sets that are now attainable from nonmodel taxa through current genomic sequencing technologies. We develop and test an ABC framework for model selection and parameter estimation, given histories of three-population divergence with admixture. We then explore different sampling regimes to illustrate how sampling more loci, longer loci or more individuals affects the quality of model selection and parameter estimation in this ABC framework. Our results show that inferences improved substantially with increases in the number and/or length of sequenced loci, while less benefit was gained by sampling large numbers of individuals. Optimal sampling strategies given our inferential models included at least 2000 loci, each approximately 2 kb in length, sampled from five diploid individuals per population, although specific strategies are model and question dependent. We tested our ABC approach through simulation-based cross-validations and illustrate its application using previously analysed data from the oak gall wasp, *Biorhiza pallida*.

## Introduction

Approximate Bayesian computation (ABC) has enjoyed increasing popularity as a method for model comparison and parameter estimation in population genetics since its introduction by Tavaré *et al*. (1997). Published reviews cover both a general introduction to ABC (Csilléry *et al*. 2010; Sunnåker *et al*. 2013) and technical aspects of its implementation (Marin *et al.* 2011; Blum *et al.* 2012). Briefly, ABC provides an approximation of the posterior distribution of model probabilities and/or parameter values by simulating data with parameters drawn from specified prior distributions and retaining values that produce data sets similar to the observed data. The similarity between observed and simulated data sets is measured by comparing summary statistics calculated from both types of data. Given sufficient summary statistics (i.e. statistics that capture all information in the data for a given parameter or model) and infinite simulations, the ABC posterior distribution should approach the true posterior in the limit of zero difference between summary statistics for observed and simulated data. Free from having to evaluate the likelihood function, ABC allows Bayesian inference while accommodating complex demographic models (Beaumont *et al.* 2002; Csilléry *et al*. 2010; Prado-Martinez *et al.* 2013). Recent developments and applications include hierarchical Bayesian analyses (Hickerson *et al.* 2006a, b; Bazin *et al.* 2010; Huang *et al.* 2011), machine learning regression techniques (Blum & François 2010) and empirical assessments of highly complex models in natural systems (Ilves *et al.* 2010; Singhal & Moritz 2012; He *et al.* 2013; Robinson *et al.* 2013).

Major challenges in ABC include the selection of sufficient summary statistics (which may not be available for the parameters or models considered; Csilléry *et al*. 2010; Aeschbacher *et al.* 2012) and the high computational cost of simulating the model-specific data to which observed values are compared. This cost is particularly significant for genome-scale data (Sousa & Hey 2013), which are nevertheless highly attractive for demographic inference because relevant parameters are best estimated from samples of many genes (Felsenstein 2006; Li & Jakobsson 2012). Because outbred diploid genomes comprise recombining segments of DNA inherited from many ancestors (Gronau *et al.* 2011), genome-level data sets for even small numbers of individuals should capture the diversity of coalescent histories across loci that reflects population history (Lohse *et al.* 2011; Leaché *et al*. 2013; Hearn *et al.* 2014). In fact, the information content of genomic data allows inference from the smallest possible samples of one haploid individual per population, as specifically explored by Hearn *et al*. (2014). Declining sequencing costs (Pool *et al.* 2010) and development of individual barcoding methods that allow population-level sampling (Baird *et al.* 2008; Peterson *et al.* 2012) increase the feasibility of genome-level sampling of nonmodel taxa.

The inherent loss of information associated with compressing data into summary statistics makes full-likelihood methods preferable to ABC (Robert *et al.* 2011), as these generally produce narrower confidence intervals and more accurate parameter estimates (Beaumont *et al.* 2002). Several analytical alternatives can handle genomic data sets (Sousa & Hey 2013) including the summary statistic-based ABBA–BABA test (Durand *et al.* 2011) to discriminate admixture from incomplete lineage sorting (Pickrell & Pritchard 2012; Eaton & Ree 2013), composite likelihood methods that exploit the site frequency spectrum (SFS; Gutenkunst *et al.* 2009; Lukić *et al*. 2011; Lukić & Hey 2012; Excoffier *et al.* 2013) and full-data genealogy sampling approaches that estimate parameters of the widely used isolation with migration (IM) model (Wang & Hey 2010). Similarly, the likelihood-based methods of Lohse *et al*. (2011) and Yang (2010) allow analysis of individual genomes collected from each of up to three populations to compare models of divergence with gene flow. The Lohse *et al*. method has been applied to study secondary contact among refugial populations (Hearn *et al.* 2014) and admixture between species (Lohse & Frantz 2014).

An important feature, though, of several likelihood-based methods (e.g. Wang & Hey 2010; Yang 2010; Lohse *et al.* 2011) is that they currently require knowledge of the ancestral state for variable sites to identify shared derived alleles between pairs of populations. It is otherwise impossible to distinguish shared high-frequency-derived alleles from high-frequency ancestral-state alleles, a distinction that can help discriminate models of post-divergence gene flow from incomplete lineage sorting (e.g. ABBA–BABA test; Durand *et al.* 2011) and help estimation of the timing and magnitude of gene flow between populations (e.g. Gutenkunst *et al.* 2009; Lukić & Hey 2012).

Further, despite their computational efficiency and use of the full data set, methods such as Lohse *et al*.'s are presently limited to analysis of haploid or phased diploid genomes for small numbers of individuals. Thus, for a triplet of populations, the Lohse *et al*. (2011) method can currently only incorporate one individual from each population (Hearn *et al.* 2014). Such minimal sampling precludes estimation of population-level parameters (e.g. effective population size; Lohse *et al.* 2012), limiting the complexity of the demographic models that can be considered. Alternatively, composite likelihood methods that exploit the SFS (Gutenkunst *et al.* 2009; Excoffier *et al.* 2013) assume the data comprise independent (i.e. unlinked) single nucleotide polymorphisms (SNPs), an unrealistic assumption for most genomic data sets that prevents such methods from exploiting information derived from linkage (e.g. Pool & Nielsen 2009).

Given current limitations of alternatives, ABC remains attractive for analysis of genome-scale data sets due to its simplicity, flexibility and ability to accommodate complex models (François *et al*. 2008; Wollstein *et al.* 2010; Li & Jakobsson 2012; Nadachowska-Brzyska *et al.* 2013; Prado-Martinez *et al.* 2013; Roux *et al.* 2013). Here, we introduce and test an ABC method to study population divergence and speciation that avoids these limitations by allowing the analysis of unphased diploid data sets for multiple individuals per population, without the need for outgroup identification of ancestral states. Our approach imposes no sampling limits on the number of populations or individuals, allowing population-level parameters (i.e. local *N*_*e*_) to be incorporated and estimated. We investigate the utility of ABC for demographic inference from population genomic data, using simulation-based validations to examine the influence of sampling attributes of the data set (number and length of loci, number of individuals) on model selection and parameter estimation. We also apply our ABC framework to a population genomic data set (Hearn *et al.* 2014) generated specifically for application of the likelihood-based method of Lohse *et al*. (2011) and compare the results of the two approaches. Our study demonstrates the promise of ABC when applied to population genomic data sets and provides sampling strategy recommendations for future studies.

## Materials and methods

### Models

**Figure 1:**
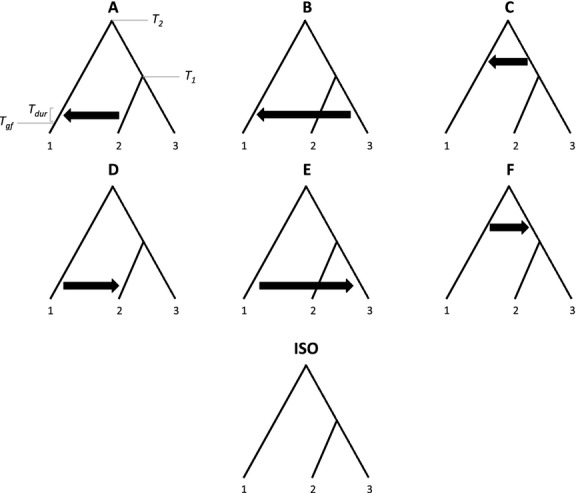
Candidate set of simulated models. Model parameters included the subpopulation scaled mutation rate (θ), the split times between populations (*T*_*1*_ and *T*_*2*_), the magnitude of gene flow during admixture (4*Nm*), the timing of gene flow (*T*_*gf*_) and its duration (*T*_*dur*_).

Our ABC approach uses data simulated under seven multi-population divergence models with post-divergence admixture between pairs of populations modelled as a continuous process over a specified time window (Fig.1). Our models are limited to three populations, but the approach is extendable to any number. The simulated models included up to six parameters: scaled subpopulation diversity (θ_*S*_ = 4*N*_*e*_μ*L*, where μ is the per base pair rate of mutation and *L* is the length of the locus), rate of gene flow during the period of admixture (*4Nm*), the time in the past at which gene flow ceased (*T*_*gf*_), the duration of admixture (*T*_*dur*_) and the timing of population divergence events (*T*_*1*_ and *T*_*2*_).

### Summary statistics

Coalescent simulations and per locus summary statistics were simulated and calculated in msABC (Pavlidis *et al.* 2010). The statistics were based on the distributions (across loci) of the four mutually exclusive categories of segregating sites in two populations (Wakeley & Hey 1997): specifically, the proportion of segregating sites categorized as fixed differences, shared polymorphisms and private polymorphisms in each pairwise population comparison. We also recorded the number of sites segregating in each population individually and in the total combined sample. The resulting 13 statistics per locus are similar to those used successfully in recent ABC analyses of population genomic data (Ross-Ibarra *et al.* 2008, 2009; Roux *et al.* 2011, 2013; Nadachowska-Brzyska *et al.* 2013; Prado-Martinez *et al.* 2013). Here, we use the first four distribution moments for each statistic across loci, giving 52 summary statistics for ABC model selection and parameter estimation. We chose moments over quantiles because of expected colinearity among quantiles calculated from the same distribution, and the invariance across loci for 0th and 100th percentiles of the distributions of percentage-based statistics. Low numbers of segregating sites per locus also resulted in particularly strong correlations among quantiles for distributions of these statistics (data not shown).

Summary statistics were calculated in R (R Development Core Team 2008) using core functions and the ‘psych’ package (Revelle 2013). To reduce the dimensionality of our 52 summary statistics (Blum *et al.* 2012), we applied the neural network method of Blum & François (2010) for parameter estimation in both simulated and empirical data sets. We further examined the influence of the number of summary statistics used by testing the performance of ABC-based model selection and parameter estimation using only the distribution means and not the higher moments, for two of the simulated sampling schemes.

### Simulation study

We used ‘pseudo-observed data set’ (PODS) experiments to assess the influence of alternative sampling schemes on parameter estimation and correct model identification. This is essential to identify how well an ABC method approximates model posterior probabilities given summary statistics that may be insufficient for model comparisons (Robert *et al.* 2011). Such approaches are often implemented a posteriori to assess the robustness of ABC conclusions (Barrès *et al*. 2012; Roux *et al.* 2013); here, we apply them to assess our ABC framework and to characterize the influences of sampling attributes on the accuracy of model choice and parameter estimation.

We simulated data under 14 sampling strategies, varying in number of diploid individuals sampled per population (1–50), locus number (200–10 000) and locus length (200 bp – 5 kb) (Table S1, Supporting information). We did not examine all possible combinations of sampling attributes; the origin of the axes we did explore centred around data sets comprising 1000 loci, each 500 bp long, for one diploid individual in each of three populations. We note that varying locus length across simulations equates to varying mutation rates or effective population sizes, as these parameters all contribute to the scaled population mutation rate parameter (θ). Our alternative sampling schemes differed in the total number of SNPs in the data set (Table S1), which increased when sampling more loci, longer loci or more individuals.

Our simulations assumed uniform mutation and introgression rates across the genome, no recombination within or linkage between loci, and equal and constant effective population sizes. While ignoring recombination within loci is common in population genetics (e.g. Beerli & Felsenstein 1999; Nielsen & Wakeley 2001), this practice can lead to estimator bias. Hearn *et al*. (2014) used simulations with varying recombination rates to show that, while biases in parameter estimates were introduced as the recombination rate surpassed the mutation rate, the correct model of population history was still recovered by their likelihood-based analysis. Further, analyses of data sets with loci trimmed to 2 kb, 1 kb and 500 bp all supported the same model and produced similar parameter estimates, indicating that undetected recombination within loci of these lengths did not severely bias their parameter estimates. Our simulated sequence lengths span the range used by Hearn *et al*. and extend to 5 kb. Although results at this upper limit may be unreliable for organisms with high recombination rates, researchers should be able to choose sequencing strategies that provide locus lengths and numbers that minimize impacts of recombination and linkage for their target organism(s).

Our PODS cross-validation experiments simulated 200,000 random prior draws from each of the seven models in Fig.1 (1.4 million data sets per sampling scheme, 19.6 million across all 14). Parameter prior distributions were identical across models (Table1). Priors for θ assumed a mutation rate of 1.75 × 10^−9^, half that estimated for *Drosophila melanogaster* (Keightley *et al.* 2009) to match Hearn *et al*. (2014), and include effective population sizes from 2000 to 100,000. All analyses were conducted using the ‘abc’ R package (Csilléry *et al*. 2012). To assess model selection performance, we used 100 ‘leave one out’ cross-validation replicates per model, wherein a single simulated data set was removed from the reference table and used as observed data. To estimate posterior model probabilities for these PODS, we used the multinomial logistic regression method (Beaumont 2008), with tolerance set to 0.1% (1400 retained data sets). For each model and sampling strategy combination, we recorded the mean posterior probability across PODS and the proportion of replicates where the true model received strong support (Bayes factor >10 in pairwise comparisons with competing models; Jeffreys 1961). Bayes factors for the latter measure of support were calculated as the posterior probability of the true model divided by that for the model with the highest posterior probability from the remaining candidates.

**Table 1:** Prior distributions used to simulate data sets for the present study (U – uniform distribution, E – exponential distribution). Theta is specified assuming a sequence locus of 500 bp for the simulation study and 1000 bp for the *Biorhiza pallida* analysis

Model parameter	Prior distribution (simulations)	Model parameter	Prior distribution (*B. pallida*)
θ	U(0.007–0.35)	θ	U(0.01–1.4)
*T*_*1*_	U(0.4–1 | > *T*_*gf*_)	*T*_*1*_	U(0.1–4 | T_1_ > T_gf_)*
*T*_*2*_	U(1–4)	*T*_*2*_	U(0.1–4 | T_2_ > T_1_)*
*T*_*gf*_	U(0.1–0.5)	*T*_*gf*_	U(0.1–2)
*T*_*dur*_	U(0.01–0.1)	*F*	U(0–1)
*Nm*	E(0.1)		

* Distribution given is for models with recent admixture (A, B, D and E). For models of ancient admixture (C and F), *T*_1_ was U(0.1–4)*, T*_2_ was U(*T*_1_ – 4) *and T*_*gf*_ was U(*T*_1_ – *T*_2_).

To assess the quality of parameter estimates resulting from our ABC approach, we used ABC to estimate parameter values for PODS simulated under four of the competing models (‘A’, ‘C’, ‘D’ and ‘ISO’), recognizing that our full set of models are inherently related in pairs (e.g. model D and E differ only in the identity of the population receiving migrants from population 1, similar pairs are AB and CF; Fig.1). Our approach incorporates one model from each pair. For each sampling scheme and model, we simulated 100 PODS by randomly drawing parameter values from the prior distributions used to generate the ABC reference table (Table1). Parameter posterior distributions were estimated using the neural network method in the ‘abc’ R package (Csilléry *et al*. 2012), with tolerance set to 0.5% (1000 retained data sets). We then calculated the prediction error (ε) for each parameter under each sampling scheme and compared the observed prediction error to that expected based on its prior distribution (see Appendix S1, Supporting information). For further assessment of the quality of parameter estimates obtained by our ABC approach, we assessed the coverage property (Prangle *et al.* 2014) and widths of 95% highest probability density (HPD) intervals of the estimated posterior distributions for each parameter.

### Empirical application

As an empirical application of our approach, we analysed genome-level data for an oak gallwasp (*Biorhiza pallida*) (Hearn *et al.* 2013, 2014), sampled from three regional populations (Iran, the Balkans and the Iberian peninsula) spanning the Western Palaearctic (Rokas *et al.* 2001). Previous work suggests that gallwasp communities, along with their *Quercus* hosts, were restricted to these southern refugia during Pleistocene glacial maxima (Stone *et al.* 2002; Rokas *et al.* 2003). The full data set of Hearn *et al*. (2014) comprised two haploid males from each of the Balkans and Iberia and one male from Iran (Fig. S1, Supporting information), each sequenced to <2-fold coverage (see Hearn *et al*. for a pipeline allowing generation of appropriate sequence loci from de novo genome sequence). To facilitate comparisons between our ABC results and those using maximum likelihood in Hearn *et al*. (2014), we reduced locus lengths to 1 kb, but instead of using a single individual per refuge, as in Hearn *et al*. (2014), we included all five individuals to make use of within-population diversity information in our ABC analysis. This configuration resulted in a total of 1203 alignable loci (from the 2231 loci analysed in Hearn *et al.* 2014). Pooling individuals from separate sites within refugia is justified by the demonstration by Hearn *et al*. that data sets for different individuals collected from the Iberian and Balkan refugia supported the same model of population history and produced similar parameter estimates.

Our ABC analysis also employed the best-supported three-population topology identified by Hearn *et al*. (2014). Although previous studies have favoured an eastern origin for members of the oak gall community (Rokas *et al.* 2003; Stone *et al.* 2007, 2009), the analysis by Hearn *et al*. (2014) unexpectedly supported older divergence of the Iberian population and more recent divergence between Balkan and Iranian populations. Despite substantial reduction in the number of aligned sequence loci in our data set, the dominant class of SNPs in the five-individual data set still grouped the Balkan and Iranian populations together, to the exclusion of the Iberian samples (Table S2, Supporting information). We therefore limited our analysis by comparing seven models, similar to those depicted in Fig.1, instead of all 21 possible model x topology combinations (as in Hearn *et al.* 2014). Models were modified slightly from those shown in Fig.1 to facilitate direct comparisons with the results obtained by Hearn *et al.* (2014). Specifically, we simulated admixture as an instantaneous event, thus replacing the duration (*T*_*dur*_) and rate (*Nm*) of gene flow with a single parameter, the admixture proportion (*F*).

Because our summary statistic strategy required more than one sequence per locus per population, our empirical analysis of the *B. pallida* data set employed fewer summary statistics calculated using the single haploid individual sampled from Iran (the putative Eastern refuge). Specifically, our empirical application employed a total of 40 summary statistics (Table S3, Supporting information), due to the lack of information on segregating sites in, and shared polymorphisms with, the Iranian population. Simulations for the empirical application in *B. pallida* were conducted using a modified version of msABC (Pavlidis *et al.* 2010). Using these 40 summary statistics, we obtained the approximate posterior probabilities of the seven models and posterior distributions for parameters of the most probable model. The prior distributions for this analysis (Table1) are based on biological knowledge of the system and span the likelihood estimates of Hearn *et al.* (2014). To better report uncertainty in the model posterior probabilities, we conducted model comparisons using a range of tolerances that accepted between 1000 and 10 000 data sets from the simulation reference table. We used both simple rejection and multinomial logistic regression (Beaumont 2008) methods for model selection, and the neural network method for parameter estimation (with a tolerance of 0.1%, 2000 retained simulations). To more fully explore posterior distributions, we simulated two million data sets per model.

Prior to model selection and parameter estimation, we used a principal components analysis (PCA) of summary statistics for 50 000 simulations per model to check that model priors were properly specified and could generate summary statistics similar to those calculated from the observed data. We used the ‘prcomp’ function in R (R Development Core Team 2008) to graphically verify that observed summary statistics clustered with the reference table entries for the simulated data sets. Following model selection, we used PCA with 1001 posterior predictive simulations (Gelman *et al.* 2003), with the same combinations of models and parameter values used to simulate accepted data sets, to compare the fit of the models receiving posterior support.

## Results and discussion

As expected, both locus length and number influenced ABC performance in model selection and parameter estimation. In contrast, inference accuracy showed relatively minor improvement when sampling more individuals. These results match previous studies showing improvements in parameter estimation with larger numbers of loci (e.g. Felsenstein 2006; Li & Jakobsson 2012). Our consideration of locus length, number and number of sampled individuals provides general sampling guidance for those seeking to apply ABC to compare models of post-divergence gene flow.

Our ABC approach is extremely flexible, requiring no ancestral-state information or phasing of alleles. Furthermore, in principle, it is extendable to more than three populations and greater model complexity, including variation in local *N*_*e*_ among populations, population expansion after divergence or multiple periods of admixture. However, further simulation-based validations beyond the scope of this study are necessary to assess performance of this framework for more parameter-rich models. We focused deliberately on simpler models for which likelihood-based analytical methods are already available (Lohse *et al.* 2011), allowing us to compare likelihood-based (Hearn *et al.* 2014) and ABC-based results for the same system. Below, we discuss our findings in terms of the two separate goals of model selection and parameter estimation and summarize results of our empirical analysis of genomic data for Western Palaearctic populations of *B. pallida*.

### Model selection

**Figure 2:**
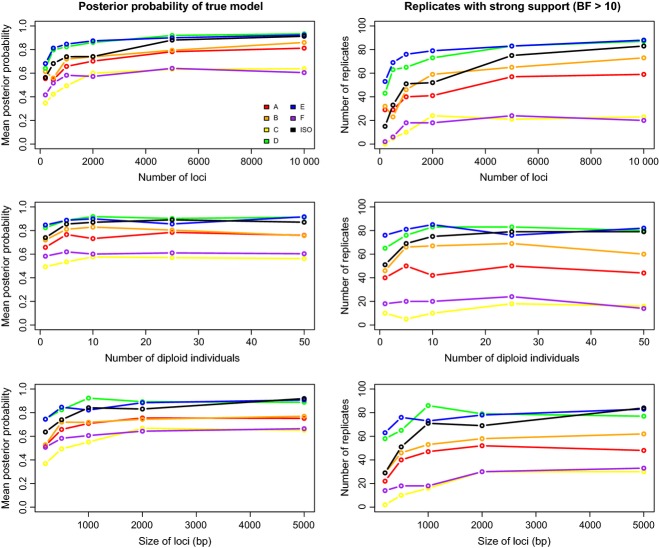
Results for model selection analyses. Plots in the left-hand column give the mean posterior probability of the true model for different sampling designs. Panels in the right-hand column show the number of replicates (out of 100) where the minimum pairwise Bayes factor in favour of the true model was >10.

Mean posterior probability of the true model and the number of replicates strongly supporting the true model increased with increasing locus size, locus number and the number of diploid individuals sampled (Fig.2). However, these sampling aspects varied in their impact on model selection. The mean posterior probability of the true model increased sharply as locus number increased from 200 to 2000, and as locus size increased from 500 bp to 2 kb, but more modestly with increasing numbers of individuals (Fig.2). Most of the gain in posterior probability for the simulated model was realized with samples as small as five diploid individuals (Fig.2). The increase in confidence associated with sampling five *versus* one diploid individual per population was sometimes substantial; mean posterior probabilities of the true model were 0.036–0.113 higher for samples of five vs. one individual. The proportion of the 100 cross-validation replicates strongly supporting the true model showed a similar relationship (Fig.2). Comparing simulations for the smallest and largest values of each sampling parameter, the average (across models) number of data sets strongly supporting the simulated model increased by 22.5% (individuals), 92.2% (locus length) and 148.9% (locus number) (Fig.2).

**Figure 3:**
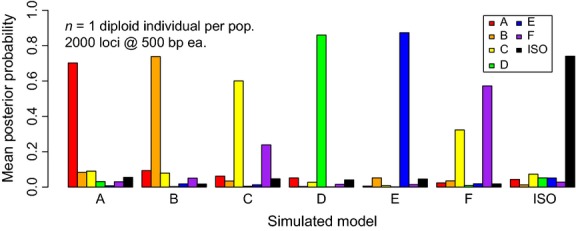
Example of mean posterior model probabilities. Results are shown from one of our simulated sampling strategies (2000 loci, 500 bp, single diploid individual per population). The *x*-axis shows the true (simulated) model, and each bar gives the mean posterior probability for that model across 100 replicates.

Cross-validation revealed inherent differences in the identifiability of the seven simulated models. Models D and E were consistently the easiest to identify, and models C and F the most difficult. These results are intuitive, as models D and E include migration from the more diverged population into one of the two more closely related populations. Such migration does not homogenize the diverged population with both sister populations. In contrast, for models A and B, migration in the opposite direction reduces genetic divergence between the diverged population and both derived sister populations due to the latters’ shared ancestry, reducing the signal available for model discrimination. Models C and F are only distinguished by a difference in the direction of admixture predating the divergence of the sister populations (Fig.1). Misclassification errors for these models were typically with respect to the direction of admixture while being correct about its timing: that is, model C data sets that were misclassified were mostly ascribed to model F and vice versa (see Fig.3 for an example).

### Parameter estimation

**Figure 4:**
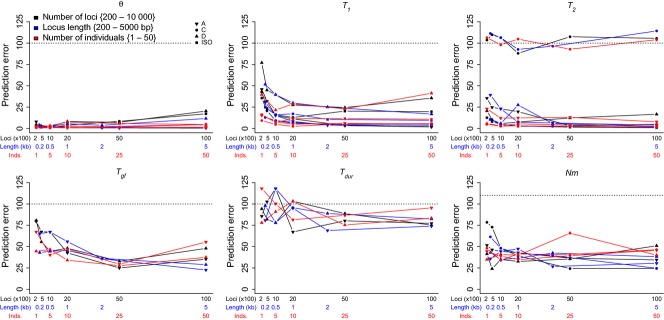
Variation in parameter prediction error with changes in sample design. Results are plotted separately for the four models (symbols) and for the three different sampling aspects varied (colours). The dotted line gives the expected prediction error based on the prior distribution.

Prediction errors for parameter estimation declined with increasing locus number and length (Figs4 and S6–S13, Supporting information), while the number of individuals sampled had relatively little effect (Figs4 and S2–S5, Supporting information). Most of the improvement occurred as locus number or size increased from the smallest to intermediate values. Thus, as for model selection, improvements with increasing sample size were subject to diminishing returns. Across the simulated models and their parameters, there was little decrease in parameter prediction error for samples of more than 2000 loci (locus length held constant at 500 bp). Similarly, locus lengths above 2 kb rarely led to large decreases in prediction error (Fig.4).

The coverage of the 95% HPD intervals was greater than 80% across all parameters, simulated models and sampling schemes considered (Table S4, Supporting information). However, in cases where no information is available for parameter estimation, the posterior matches the prior distribution, and coverage of the HPD intervals would be 95%. Therefore, we also examined the widths of the 95% HPD intervals to determine whether the confidence in a given parameter estimate increased with changes in the sampling strategy. Generally, 95% HPD interval widths declined with increasing locus length and number, but not with increasing numbers of individuals (Figs S14–S16, Supporting information). Several model parameters showed no improvement in HPD interval width with increased sampling, and these parameters were specific to particular models. For instance, duration of gene flow (*T*_*dur*_) in models A and D consistently produced 95% HPD intervals that were nearly as wide as the prior distribution. The splitting time between populations 1 and 2 (*T*_*2*_) in model C had similarly wide 95% HPD intervals. Both parameters (*T*_*dur*_ and *T*_*2*_ in model C) show prediction errors centred on that expected based on the prior distribution (Fig.4). Most of the reduction in the interval width was achieved by sampling ≥1000 loci ≥1 kb in length. However, further improvement in HPD intervals for the split times (*T*_1_ and *T*_2_) was apparent in data sets of 5000 or more loci (Fig. S15, Supporting information). For the largest sample sizes, many parameters had 95% HPD intervals that were ∼¼ the width of the prior distribution. Further improvement in parameter estimates might be possible if locus number and length were increased simultaneously. For instance, samples of 2000 loci, each 2 kb in length, might yield better estimates of parameters and/or tighter HPD intervals than any of these sampling schemes.

The relative accuracies of parameter estimates were also model dependent (Fig.4). As noted above, prediction error for *T*_2_ was largest in model C, where little information was available for parameter estimation due to admixture between *T*_1_ and *T*_2_. With this exception, prediction errors for θ, *T*_1_ and *T*_2_ were generally small for large sample sizes. In contrast, parameters associated with admixture were difficult to estimate, with all three parameters (*T*_*gf*_, *T*_*dur*_ and *Nm*) showing high prediction error (Fig.4). These results agree with previous work showing that the SFS alone is insufficient to accurately infer timing of admixture between populations (Sousa *et al.* 2011; Strasburg & Rieseberg 2011). In future work, estimates of geneflow timing may be improved by accounting for recombination and linkage disequilibrium, perhaps using information on the sizes of migrant sequence blocks (Pool & Nielsen 2009) or of regions of identity-by-descent surrounding shared derived SNP alleles (Theunert *et al.* 2012).

### Impacts of summary statistic reduction on model choice and parameter estimates

For two sampling schemes (1000 and 10 000 loci, each 500 bp long, sampled from 1 diploid individual per population), we assessed the performance of ABC model selection and parameter estimation using only the means of the distributions of statistics (13 total statistics). Mean posterior probabilities for the true model, and the number of strongly supported replicates, were highly concordant with those obtained using the full set of 52 summary statistics (Figs S17 and S18, Supporting information). This suggests that, for these models, the statistic means contain most of the available information for model selection. Prediction errors for parameters were also generally comparable between analyses using all 52 statistics and the reduced set of 13 statistics. However, all parameters of the isolation model and model D in the larger simulated data sets (10 000 loci) had substantially lower prediction errors when using the reduced set of 13 statistics (Figs S19–S22, Supporting information). Thus, in some cases, alternative sets of summary statistics may provide more robust inference under our analytical framework.

### Sampling strategies

Our results suggest that an effective and cost-efficient population genomic data set for comparing models of secondary contact and admixture would include many loci (∼2000) of intermediate length (∼2 kb) sampled from relatively few individuals (∼5). We stress that these recommendations are specific to the models compared, and the time frame of divergence and admixture modelled here. However, the models we examine are general, and many species exposed to cyclical climatic changes in the Pleistocene (e.g. Pleistocene ‘breathing’ models; Jesus *et al.* 2006) may have experienced admixture on time frames matching our simulations. Furthermore, Li & Jakobsson (2012) found that similar numbers of much larger loci (1000–2000 loci, each 100 kb long) were sufficient for accurate parameter estimates in two-population divergence models, suggesting that our results may apply more broadly.

As a *post hoc* assessment of our recommended sampling strategy, we conducted additional PODS simulations with data sets composed of 2000 loci, each 2 kb in length, sampled from 5 diploid individuals per population (a sampling strategy not explicitly considered in our simulation study). The performance of these data sets for both model selection and parameter estimation was assessed as above. Model selection cross-validations support our recommended sampling scheme. Results of these analyses were similar to those seen in previous simulations, with models C and F showing the lowest posterior probabilities and fewest replicates with strong support (Table S5, Supporting information). Nonetheless, both measures of model selection performance (mean posterior probability and the number of highly supported replicates) indicated that our recommended sampling scheme performed as well as, or better than, the largest data sets considered. Likewise, prediction errors for the parameters of models A, C, D and ISO given our optimal sampling scheme were similar to those calculated for data sets that included 10 000 loci (Table S6, Supporting information).

### Empirical application

ABC analysis of the *B. pallida* data set gave results comparable to those obtained by Hearn *et al.* (2014). However, our ABC approach resulted in substantially more uncertainty, particularly in model comparisons. Using data sets simulated for the ABC reference table, we verified that our prior distributions were capable of generating data resembling those observed (Fig. S23, Supporting information). Posterior probabilities from the ABC analysis using simple rejection consistently supported models A, B, C and F above the remaining models across the range of tolerances examined (Table2). In contrast, multinomial logistic regression (Beaumont 2008) returned idiosyncratic model posterior probabilities that differed substantially from those obtained with simple rejection (Table2). Given the consistency of rejection-based model probabilities across tolerances, and the observation that narrower tolerances led to increased support for the same model (B) supported in Hearn *et al.* (2014) (Table2), we focus our parameter estimation analyses on the four models (A, B, C and F) best supported by the simple rejection method.

**Table 2:** Posterior probabilities of the seven candidate models when compared in the *Biorhiza pallida* system. Results are presented for a) the rejection method and b) the multinomial logistic regression method with between 1000 and 10 000 accepted data sets. Posterior probabilities for the best model in each case are given in *bold italics*

Data sets accepted	A	B	C	D	E	F	ISO
Rejection method							
1001	0.2298	***0.2977***	0.1459	0.0220	0.0360	0.2028	0.0659
5000	0.2288	***0.2370***	0.1800	0.0416	0.0362	0.1880	0.0884
10 000	***0.2577***	0.2094	0.1759	0.0626	0.0310	0.1784	0.0850
Multinomial Logistic Regression							
1001	0	0	0	0	*1*	0	0
5000	0.0012	0.0244	0.0994	0.0001	0.0059	***0.8681***	0.0009
10 000	0.0024	0.0202	0.0679	0.0003	0.0023	***0.9034***	0.0034

**Figure 5:**
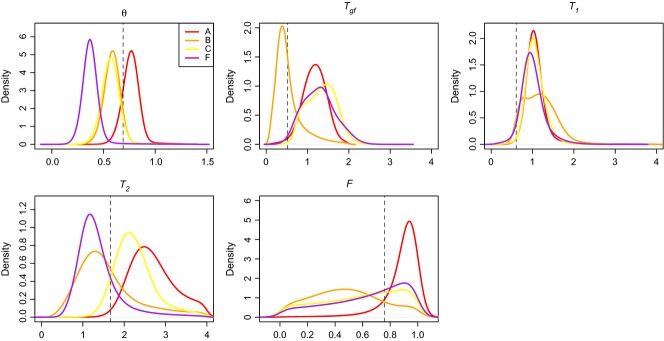
Parameter posterior distributions for data from *Biorhiza pallida*. Posteriors for the four best-supported models are plotted. Point estimates obtained from the full-likelihood analysis of Hearn *et al.* (2014) are shown as vertical dashed lines. Priors were uniform, except in the case of timing parameters (*T*_*gf*_, *T*_1_ and *T*_2_), which were constrained as shown in Table1.

Despite uncertainty in model selection, parameter posterior distributions estimated via ABC were surprisingly consistent across models, suggesting that conditional model averaging may be fruitful (Table3 and Fig.5). Parameter estimates from all models suggest relatively close correspondence between the timing of gene flow (*T*_*gf*_) and the divergence between the more easterly populations (*T*_1_), consistent with Hearn *et al.* (2014). Overall, posterior distributions for model B parameters were also consistent with likelihood-based estimates. Our posterior distributions suggest a slightly lower θ, higher *T*_1_ and lower *T*_2_, but agree closely with likelihood-based estimates of *T*_*gf*_ (Fig.5). In contrast, the posterior distribution for *F* for model B resembles the prior distribution, indicating that our statistics contain little information for its estimation. It is notable that our ABC assessment of phylogeographic history in *B. pallida* required substantially more computational time than the likelihood analysis of the same data in Hearn *et al*. (see Appendix S1, Supporting information).

**Table 3:** Parameter estimates and associated 95% HPD intervals for *Biorhiza pallida*. Estimates are based on the neural network method with a tolerance of 0.1% (2000 accepted simulations). Point estimates reported are the medians of the posterior distributions

Parameter	A	B	C	F
θ	0.6203 {0.5426–0.7198}	0.4391 {0.0708–1.2554}	0.6076 {0.4985–0.7203}	0.4474 {0.3746–0.5226}
*T*_*gf*_	0.8164 {0.5045–1.1183}	0.5380 {0.1396–1.0557}	0.9220 {0.6466–1.1885}	1.0008 {0.7208–1.2561}
*T*_*1*_	1.0014 {0.8266–1.2156}	1.0187 {0.6447–1.5286}	0.8115 {0.5301–1.0040}	0.8523 {0.6870–0.9958}
*T*_*2*_	3.5226 {2.9361–3.9273}	2.2543 {1.0271–3.8996}	2.6666 {2.0141–3.6708}	2.8332 {2.2730–3.7182}
*F*	0.9496 {0.7496–0.9925}	0.6333 {0.1159–0.9819}	0.6452 {0.0909–0.9806}	0.8719 {0.1953–0.9966}

The *B. pallida* data set was outside of the specific sampling designs we considered in our simulation study and thereby highlights limitations of our approach when faced with minimal sampling. The single Iranian individual reduced the number of available summary statistics for our analysis. We speculate that both the minor discrepancies in parameter estimates between the two analyses and the uncertainty in ABC model selection reflect the combined effects of using a slightly different data set (more individuals and fewer loci) and a reduced set of summary statistics (specifically, inability to identify shared polymorphisms between the Iranian refuge and more westerly populations). Our simulation results suggest that more accurate inferences might be gained from larger numbers of longer loci sampled from multiple individuals per population. Importantly, the subset of loci employed for our empirical analysis still has a majority of informative SNPs supporting the topology favoured in Hearn *et al.* (2014), where the Iranian individual is more closely related to individuals sampled in the Balkans (Table S2).

While we have not considered all possible models of demographic history in *B. pallida*, the relatively simple models we explore demonstrate the feasibility of the ABC methodology for large genomic-scale data sets. These data can now be collected for nonmodel taxa within realistic budget constraints. The bioinformatics pipeline for whole-genome shotgun sequencing introduced in Hearn *et al.* (2014) outlines generation of suitable population genomic data in nonmodel systems. Hearn *et al.* (2014) produced a meta-assembly from de novo low-coverage genomic data of five gall wasp individuals and used it to generate alignments of >2000 orthologous loci, each longer than 2 kb. For another example using reduced representation libraries (in *Sceloporus* spiny lizards), see Leaché *et al*. (2013).

A key feature of the ABC framework is that it allows comparison of more complex models. As long as summary statistics exist that capture differences in such models, this represents a major advantage over likelihood-based analyses. For instance, several previous studies have found evidence for variable introgression rates among different regions of the genome, particularly in situations involving admixture between closely related species (Rieseberg *et al.* 1999; Carling & Brumfield 2009; Roux *et al.* 2013; Fraïsse *et al*. 2014). Although methods are available to incorporate this variation in models of divergence with gene flow (Sousa *et al.* 2013), our models assumed a constant rate of introgression for all sampled loci. If the barrier to gene flow has been stronger in some genome regions in the *B. pallida* system, our analysis would result in biased estimates for parameters associated with admixture. However, this bias may be minimized by the relatively shallow divergence between refugial populations of *B. pallida* (<200 ky), as selection against introgression is unlikely to be widespread in the genome given the recent nature of divergence among these populations.

## Conclusions

Our simulation study shows the potential of ABC for inference of population history from genomic data for small population samples. Quality of inference (for both model selection and parameter estimation) improved with increasing numbers and lengths of aligned sequence loci, and to a lesser extent with increasing numbers of individuals sampled per population. Advantages of this ABC approach relative to existing likelihood frameworks include (i) consideration of more complex models, (ii) relaxation of assumptions concerning the relative mutation/introgression rates across loci and the lack of recombination, (iii) analysis of larger samples from each population, and (iv) analysis of data without information on phasing of alleles or ancestral state. Our empirical application shows limitations of the ABC approach for minimal population sampling of a single individual and the importance of obtaining appropriate summary statistics for robust inference. A natural extension of this work is to consider models that include the possibility of selection, intralocus recombination, admixture that declines with time after divergence (Heled *et al.* 2013), variation across the genome in mutation or introgression rates (Roux *et al.* 2013), dynamically changing effective population sizes in refugial populations or multiple episodes of admixture, as might be driven by cyclical climatic oscillations during the Pleistocene (Jesus *et al.* 2006).

## Data accessibility

All simulation and analysis scripts associated with our project are available on DRYAD at http://datadryad.org/resource/doi:10.5061/dryad.80m5b. Additionally, the raw oak gall wasp data are available at http://datadryad.org/resource/doi:10.5061/dryad.r3r60.

## Supporting Information

Table S1 Sampling strategies considered during our simulation study. **Table S2** Counts of the number of single nucleotide polymorphisms (SNPs) that support different groups in the empirical dataset (1080 loci). **Table S3** Observed values of the 40 summary statistics used for the ABC application to data from *Biorhiza pallida*. **Table S4** Coverage of the 95% HPD intervals estimated for pseudo-observed datasets (PODS). **Table S5** Mean posterior probabilities, and the number of replicates with strong support (minimum Bayes Factor > 10), for the simulated model under the “optimal” sampling strategy. **Table S6** Prediction errors for model parameters under the “optimal” sampling strategy vs. the largest datasets simulated varying the number of individuals, the number of loci, or the lengths of loci.

Fig S1 Sampling locations for the *Biorhiza pallida* individuals included in our empirical application. **Fig. S2** Parameter estimates for the parameters of model A (columns), plotted for datasets with different numbers of sampled individuals (rows). **Fig. S3** Parameter estimates for the parameters of model C (columns), plotted for datasets with different numbers of sampled individuals (rows). **Fig. S4** Parameter estimates for the parameters of model D (columns), plotted for datasets with different numbers of sampled individuals (rows). **Fig. S5** Parameter estimates for the parameters of model ISO (columns), plotted for datasets with different numbers of sampled individuals (rows). **Fig. S6** Parameter estimates for the parameters of model A (columns), plotted for datasets with different numbers of loci (rows). **Fig. S7** Parameter estimates for the parameters of model C (columns), plotted for datasets with different numbers of loci (rows). **Fig. S8** Parameter estimates for the parameters of model D (columns), plotted for datasets with different numbers of loci (rows). **Fig. S9** Parameter estimates for the parameters of model ISO (columns), plotted for datasets with different numbers of loci (rows). **Fig. S10** Parameter estimates for the parameters of model A (columns), plotted for datasets with different lengths of loci (rows). **Fig. S11** Parameter estimates for the parameters of model C (columns), plotted for datasets with different lengths of loci (rows). **Fig. S12** Parameter estimates for the parameters of model D (columns), plotted for datasets with different lengths of loci (rows). **Fig. S13** Parameter estimates for the parameters of model ISO (columns), plotted for datasets with different lengths of loci (rows). **Fig. S14** Widths of confidence intervals for parameters (columns) of models A, C, D, and ISO (rows), plotted separately for datasets with different numbers of sampled individuals. **Fig. S15** Widths of confidence intervals for parameters (columns) of models A, C, D, and ISO (rows), plotted separately for datasets with different numbers of loci. **Fig. S16** Widths of confidence intervals for parameters (columns) of models A, C, D, and ISO (rows), plotted separately for datasets with different sizes of loci. **Fig. S17** Results for model selection analyses for simulated datasets using all 52 summary statistics (lines) and for two sampling schemes when using only the means of the statistic distributions (“X”). **Fig. S18** Results for model selection analyses for simulated datasets using all 52 summary statistics (lines) and for two sampling schemes when using only the means of the statistic distributions (“X”). **Fig. S19** Prediction errors for parameters of model A, across datasets sampling different numbers of loci using all 52 summary statistics (lines), or only the means of the statistic distributions (“X”). **Fig. S20** Prediction errors for parameters of model C, across datasets sampling different numbers of loci using all 52 summary statistics (lines), or only the means of the statistic distributions (“X”). **Fig. S21** Prediction errors for parameters of model D, across datasets sampling different numbers of loci using all 52 summary statistics (lines), or only the means of the statistic distributions (“X”). **Fig. S22** Prediction errors for parameters of model ISO, across datasets sampling different numbers of loci using all 52 summary statistics (lines), or only the means of the statistic distributions (“X”). **Fig. S23** Principal components analyses of the simulated and observed summary statistics for the seven candidate models (A-F, ISO) and the *B. pallida* dataset.

Appendix S1 Supplementary methods: Calculation of observed and expected prediction errors for parameter estimates and computational load..
