# Supplementary material for: ABC inference of multi-population divergence with admixture from unphased population genomic data
Source: Mol Ecol. 2014 Sep 6;23(18):4458–71. doi: 10.1111/mec.12881 (PMC4285295; doi:10.1111/mec.12881)

**Supporting Table 1.**

Sampling strategies considered during our simulation study. Three attributes of the dataset were varied: the number of diploid individuals (*n*), the number of loci (*L*), and the locus lengths (*s*). We also provide the average total number of segregating sites (*S_T_*) for each sampling scheme.

| **Combination** | ***n*** | ***L*** | ***s* (bp)** | ***S_T_^*^*** |
| --- | --- | --- | --- | --- |
| 1 | 1 | 1000 | 500 | 1351.6 |
| 2 | 5 | 1000 | 500 | 2144.0 |
| 3 | 10 | 1000 | 500 | 2511.2 |
| 4 | 25 | 1000 | 500 | 2995.4 |
| 5 | 50 | 1000 | 500 | 3360.2 |
| 6 | 1 | 200 | 500 | 270.2 |
| 7 | 1 | 500 | 500 | 674.9 |
| 8 | 1 | 2000 | 500 | 2700.1 |
| 9 | 1 | 5000 | 500 | 6749.4 |
| 10 | 1 | 10000 | 500 | 13,479.6 |
| 11 | 1 | 1000 | 200 | 541.0 |
| 12 | 1 | 1000 | 1000 | 2693.7 |
| 13 | 1 | 1000 | 2000 | 5385.3 |
| 14 | 1 | 1000 | 5000 | 13,480.5 |

*: Average *S_T_* was calculated for simulations of model A.

**Supporting Table 2.**

Counts of the number of single nucleotide polymorphisms (SNPs) that support different groups in the empirical dataset (1080 loci). Group labels are as follows, W – Iberian (Western) population, C – Balkan (Central) refuge, E – Iranian (Eastern) refuge. Subscripts (a and b) denote the two individuals sampled from the Iberian and Balkan refuges.

| **Group** | **SNP Count** |
| --- | --- |
| W_a_ | 448 |
| W_b_ | 278 |
| C_a_ | 236 |
| C_b_ | 244 |
| E | 785 |
| W_a_&W_b_ / C_a_,C_b_&E | 742 |
| C_a_&C_b_ / W_a_,W_b_&E | 669 |
| C_a_&W_a_ | 6 |
| C_a_&W_b_ | 2 |
| C_a_&E | 56 |
| W_a_&E | 4 |
| C_b_&W_a_ | 0 |
| C_b_&W_b_ | 1 |
| W_b_&E | 5 |
| C_b_&E | 55 |

**Supporting Table 3.**

Observed values of the 40 summary statistics used for the ABC application to data from *Biorhiza pallida*.

| **Category** | **Statistic** | **Observed** |
| --- | --- | --- |
| Total Segregating Sites | Mean | 2.968412303 |
|  | Variance | 4.892512203 |
|  | Skew | 1.114662616 |
|  | Kurtosis | 2.19909851 |
| Segregating Sites, Iberia | Mean | 0.651704073 |
|  | Variance | 0.921018308 |
|  | Skew | 2.462628566 |
|  | Kurtosis | 9.984108924 |
| Segregating Sites, Balkans | Mean | 0.498753117 |
|  | Variance | 1.291803768 |
|  | Skew | 3.18603034 |
|  | Kurtosis | 14.67819734 |
| Proportion Shared, Iberian-Balkan | Mean | 0.001758157 |
|  | Variance | 0.000604842 |
|  | Skew | 18.03368112 |
|  | Kurtosis | 380.2048787 |
| Proportion Fixed, Iberian-Balkan | Mean | 0.521894569 |
|  | Variance | 0.161897978 |
|  | Skew | -0.095814429 |
|  | Kurtosis | -1.528825626 |
| Proportion Fixed, Iberian-Iranian | Mean | 0.654669516 |
|  | Variance | 0.142950604 |
|  | Skew | -0.658940007 |
|  | Kurtosis | -1.010014662 |
| Proportion Fixed, Balkan-Iranian | Mean | 0.762773665 |
|  | Variance | 0.146927903 |
|  | Skew | -1.185684865 |
|  | Kurtosis | -0.329357881 |
| Proportion Private, Iberian-Balkan | Mean | 0.476347274 |
|  | Variance | 0.160914114 |
|  | Skew | 0.099793117 |
|  | Kurtosis | -1.523083216 |
| Proportion Private, Iberian-Iranian | Mean | 0.345330484 |
|  | Variance | 0.142950604 |
|  | Skew | 0.658940007 |
|  | Kurtosis | -1.010014662 |
| Proportion Private, Balkan-Iranian | Mean | 0.237226335 |
|  | Variance | 0.146927903 |
|  | Skew | 1.185684864 |
|  | Kurtosis | -0.329357881 |

**Supporting Table 4.**

Coverage of the 95% HPD intervals estimated for pseudo-observed datasets (PODS). Results are separated based on the sampling aspect varied: a) the number of sequence loci sampled (locus length fixed at 500bp, number of individuals fixed at 1 diploid per population), b) the length of loci (number of loci fixed at 1000, number of individuals fixed at 1 diploid per population), and c) the number of diploid individuals collected (number of loci fixed at 1000, locus length fixed at 500 bp). A, C, D, and ISO refer to four of the seven models depicted in Figure 1.

| **(a)** |  | | | |  | | | |  | | | |  | | |  | |  | |
| --- | --- | --- | --- | --- | --- | --- | --- | --- | --- | --- | --- | --- | --- | --- | --- | --- | --- | --- | --- |
|  | ***θ*** | | | | ***T_1_*** | | | | ***T_2_*** | | | | ***Nm*** | | | ***T_gf_*** | | ***T_dur_*** | |
| **Number of Loci** | **A** | **C** | **D** | **ISO** | **A** | **C** | **D** | **ISO** | **A** | **C** | **D** | **ISO** | **A** | **C** | **D** | **A** | **D** | **A** | **D** |
| 200 | 0.91 | 0.94 | 0.95 | 0.92 | 0.87 | 0.94 | 0.96 | 0.87 | 0.91 | 0.87 | 0.94 | 0.95 | 0.94 | 0.89 | 0.97 | 0.94 | 0.94 | 0.92 | 0.95 |
| 500 | 0.95 | 0.95 | 0.95 | 0.92 | 0.93 | 0.93 | 0.92 | 0.83 | 0.91 | 0.9 | 0.99 | 0.9 | 0.94 | 0.89 | 0.93 | 0.98 | 0.94 | 0.95 | 0.92 |
| 1000 | 0.96 | 0.92 | 0.93 | 0.93 | 0.9 | 0.95 | 0.94 | 0.9 | 0.96 | 0.85 | 0.91 | 0.88 | 0.92 | 0.9 | 0.97 | 0.87 | 0.92 | 0.97 | 0.97 |
| 2000 | 0.94 | 0.99 | 0.96 | 0.89 | 0.91 | 0.93 | 0.94 | 0.87 | 0.89 | 0.92 | 0.97 | 0.9 | 0.98 | 0.94 | 0.93 | 0.97 | 0.92 | 0.93 | 0.96 |
| 5000 | 0.97 | 0.98 | 0.96 | 0.89 | 1 | 0.95 | 0.98 | 0.84 | 0.95 | 0.96 | 0.98 | 0.83 | 0.97 | 0.95 | 0.93 | 0.96 | 0.93 | 0.95 | 0.96 |
| 10000 | 1 | 0.99 | 0.93 | 0.92 | 0.96 | 0.97 | 0.94 | 0.92 | 0.95 | 0.89 | 0.92 | 0.92 | 0.92 | 0.94 | 0.96 | 0.9 | 0.96 | 0.89 | 0.94 |
| **(b)** |  |  |  |  |  |  |  |  |  |  |  |  |  |  |  |  |  |  |  |
|  | ***θ*** | | | | ***T_1_*** | | | | ***T_2_*** | | | | ***Nm*** | | | ***T_gf_*** | | ***T_dur_*** | |
| **Locus Length (bp)** | **A** | **C** | **D** | **ISO** | **A** | **C** | **D** | **ISO** | **A** | **C** | **D** | **ISO** | **A** | **C** | **D** | **A** | **D** | **A** | **D** |
| 200 | 0.96 | 0.96 | 0.94 | 0.93 | 0.92 | 0.95 | 0.94 | 0.88 | 0.91 | 0.91 | 0.95 | 0.91 | 0.98 | 0.96 | 0.94 | 0.91 | 0.97 | 0.95 | 0.92 |
| 500 | 0.96 | 0.92 | 0.93 | 0.93 | 0.9 | 0.95 | 0.94 | 0.9 | 0.96 | 0.85 | 0.91 | 0.88 | 0.92 | 0.9 | 0.97 | 0.87 | 0.92 | 0.97 | 0.97 |
| 1000 | 0.96 | 0.91 | 0.95 | 0.93 | 0.91 | 0.96 | 0.94 | 0.85 | 0.95 | 0.9 | 0.95 | 0.87 | 0.93 | 0.89 | 0.95 | 0.87 | 0.95 | 0.91 | 0.95 |
| 2000 | 0.98 | 0.94 | 0.96 | 0.96 | 0.97 | 0.94 | 0.92 | 0.87 | 0.95 | 0.89 | 0.93 | 0.94 | 0.95 | 0.92 | 0.93 | 0.91 | 0.96 | 0.88 | 0.95 |
| 5000 | 0.93 | 0.97 | 0.96 | 0.94 | 0.93 | 0.94 | 0.96 | 0.95 | 0.93 | 0.83 | 0.92 | 0.91 | 0.97 | 0.94 | 0.95 | 0.92 | 0.95 | 0.94 | 0.93 |
| **(c)** |  |  |  |  |  |  |  |  |  |  |  |  |  |  |  |  |  |  |  |
|  | ***θ*** | | | | ***T_1_*** | | | | ***T_2_*** | | | | ***Nm*** | | | ***T_gf_*** | | ***T_dur_*** | |
| **Number of Individuals** | **A** | **C** | **D** | **ISO** | **A** | **C** | **D** | **ISO** | **A** | **C** | **D** | **ISO** | **A** | **C** | **D** | **A** | **D** | **A** | **D** |
| 1 | 0.96 | 0.92 | 0.93 | 0.93 | 0.9 | 0.95 | 0.94 | 0.9 | 0.96 | 0.85 | 0.91 | 0.88 | 0.92 | 0.9 | 0.97 | 0.87 | 0.92 | 0.97 | 0.97 |
| 5 | 0.96 | 0.98 | 0.97 | 0.95 | 0.94 | 0.97 | 0.95 | 0.91 | 0.94 | 0.86 | 0.97 | 0.94 | 0.91 | 0.93 | 0.91 | 0.96 | 0.95 | 0.9 | 0.9 |
| 10 | 0.97 | 0.98 | 0.95 | 0.92 | 0.97 | 0.94 | 0.93 | 0.95 | 0.92 | 0.89 | 0.97 | 0.87 | 0.95 | 0.94 | 0.91 | 0.93 | 0.91 | 0.88 | 0.96 |
| 25 | 1 | 0.98 | 0.96 | 0.97 | 0.95 | 0.96 | 0.96 | 0.87 | 0.94 | 0.9 | 0.95 | 0.97 | 0.94 | 0.92 | 0.97 | 0.91 | 0.99 | 0.96 | 0.97 |
| 50 | 0.97 | 0.98 | 0.94 | 0.96 | 0.99 | 0.97 | 0.93 | 0.96 | 0.94 | 0.9 | 0.92 | 0.93 | 0.93 | 0.97 | 0.92 | 0.93 | 0.96 | 0.91 | 0.92 |

**Supporting Table 5.**

Mean posterior probabilities, and the number of replicates with strong support (minimum Bayes Factor > 10), for the simulated model under the “optimal” sampling strategy.

|  | Mean Posterior Probability | Replicates with Strong Support |
| --- | --- | --- |
| A | 0.834 | 57 |
| B | 0.830 | 65 |
| C | 0.701 | 30 |
| D | 0.938 | 89 |
| E | 0.935 | 85 |
| F | 0.699 | 30 |
| ISO | 0.917 | 84 |

**Supporting Table 6.**

Prediction errors for model parameters under the “optimal” sampling strategy vs. the largest datasets simulated varying the number of individuals, the number of loci, or the lengths of loci.

| Sampling Scheme | Individuals (per population) | Loci | Locus Length | Model | ε(θ) | ε(*Nm*) | ε(*T_gf_*) | ε(*T_1_*) | ε(*T_2_*) | ε(*T_dur_*) |
| --- | --- | --- | --- | --- | --- | --- | --- | --- | --- | --- |
| Individuals | 50 | 1000 | 500 bp | A | 1.372 | 45.670 | 55.299 | 5.303 | 7.761 | 95.389 |
|  |  |  |  | C | 0.896 | 46.405 | -- | 5.682 | 103.983 | -- |
|  |  |  |  | D | 4.597 | 39.948 | 37.404 | 41.296 | 4.875 | 83.301 |
|  |  |  |  | ISO | 4.707 | -- | -- | 10.752 | 1.778 | -- |
| Loci | 1 | 10000 | 500 bp | A | 0.801 | 34.398 | 35.247 | 1.751 | 4.330 | 77.188 |
|  |  |  |  | C | 0.350 | 24.362 | -- | 2.062 | 105.642 | -- |
|  |  |  |  | D | 20.340 | 50.951 | 47.886 | 35.582 | 16.628 | 75.194 |
|  |  |  |  | ISO | 17.134 | -- | -- | 19.674 | 0.905 | -- |
| Locus Length | 1 | 1000 | 5000 bp | A | 0.898 | 30.412 | 22.562 | 4.047 | 3.904 | 73.947 |
|  |  |  |  | C | 0.674 | 24.545 | -- | 3.794 | 114.321 | -- |
|  |  |  |  | D | 11.609 | 38.150 | 28.817 | 16.802 | 2.287 | 82.349 |
|  |  |  |  | ISO | 4.455 | -- | -- | 9.442 | 1.405 | -- |
| "Optimal" | 5 | 2000 | 2000 bp | A | 0.513 | 48.484 | 22.043 | 2.059 | 5.223 | 105.450 |
|  |  |  |  | C | 0.295 | 31.267 | -- | 1.461 | 86.744 | -- |
|  |  |  |  | D | 13.485 | 50.430 | 29.222 | 24.326 | 3.779 | 84.540 |
|  |  |  |  | ISO | 6.651 | -- | -- | 7.027 | 1.232 | -- |

**Supporting Figure 1.**

Sampling locations for the *Biorhiza pallida* individuals included in our empirical application. Sample labels follow Hearn *et al.* (2013). Refugial regions are colour-coded as follows: W, West, in tan; C, Centre, in orange; and E, East, in blue. The green line shows the current postglacial extent of the oak host plants (white oaks, Quercus section Quercus) exploited by *B. pallida*.


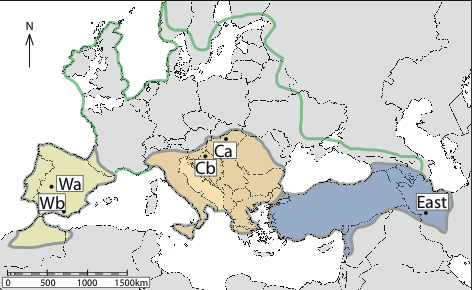


**Supporting Figure 2-13.**

Estimated parameter values vs. simulated values, for number of individuals (2-5), number of loci (6-9), and locus size (10-13). Parameters of the model are organized into columns, with rows corresponding to different sampling designs. The top row in all cases corresponds to data from the smallest sample size (in terms of number of individuals, number of loci, or locus length), and the bottom row with the largest. Plots are shown in the following order: model A, model C, model D, model ISO.

**Supporting Figure 2.**

Parameter estimates for the parameters of model A (columns), plotted for datasets with different numbers of sampled individuals (rows).


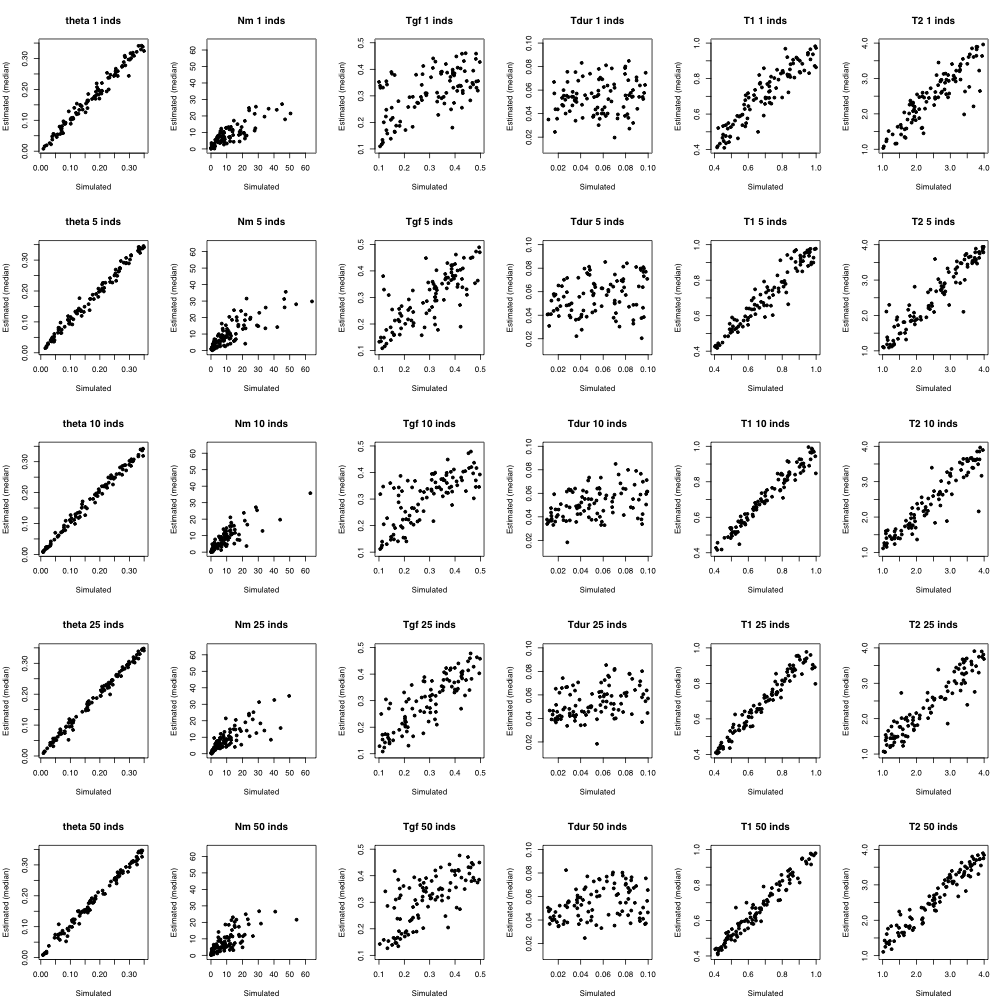


**Supporting Figure 3.**

Parameter estimates for the parameters of model C (columns), plotted for datasets with different numbers of sampled individuals (rows).


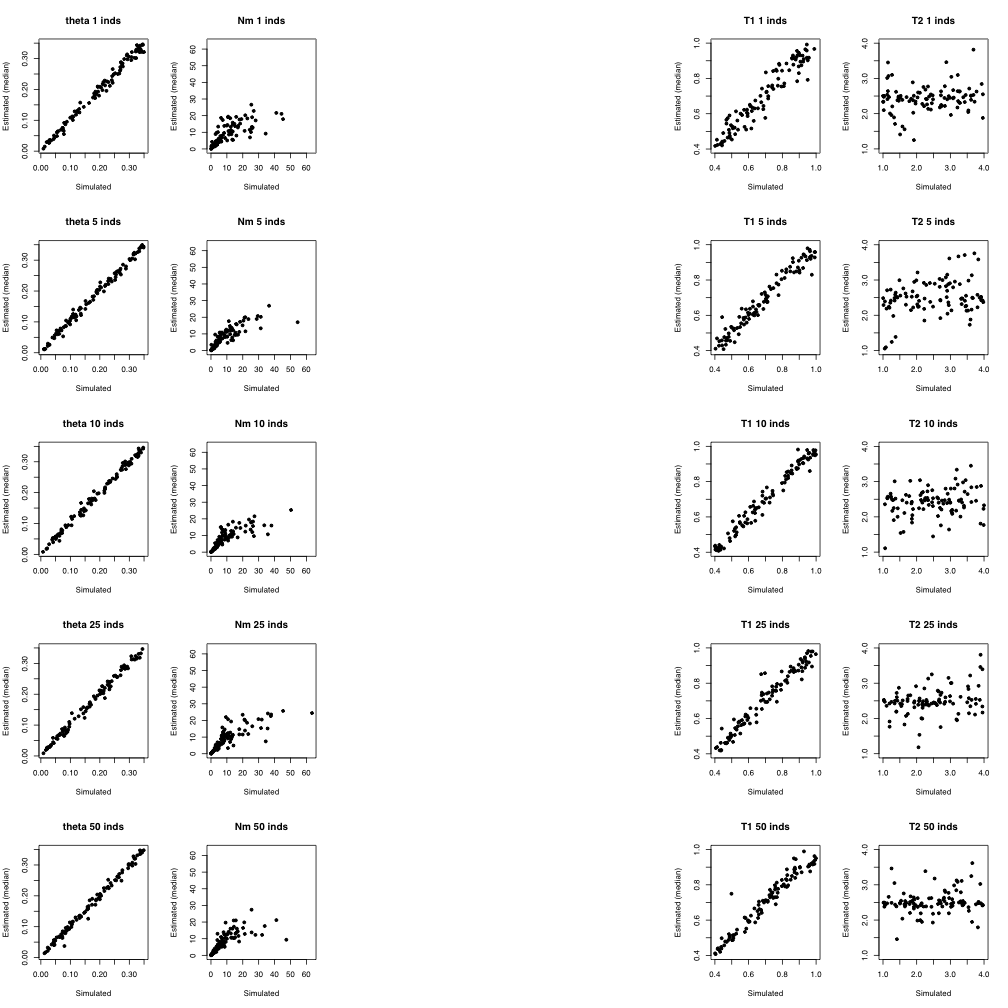


**Supporting Figure 4.**

Parameter estimates for the parameters of model D (columns), plotted for datasets with different numbers of sampled individuals (rows).


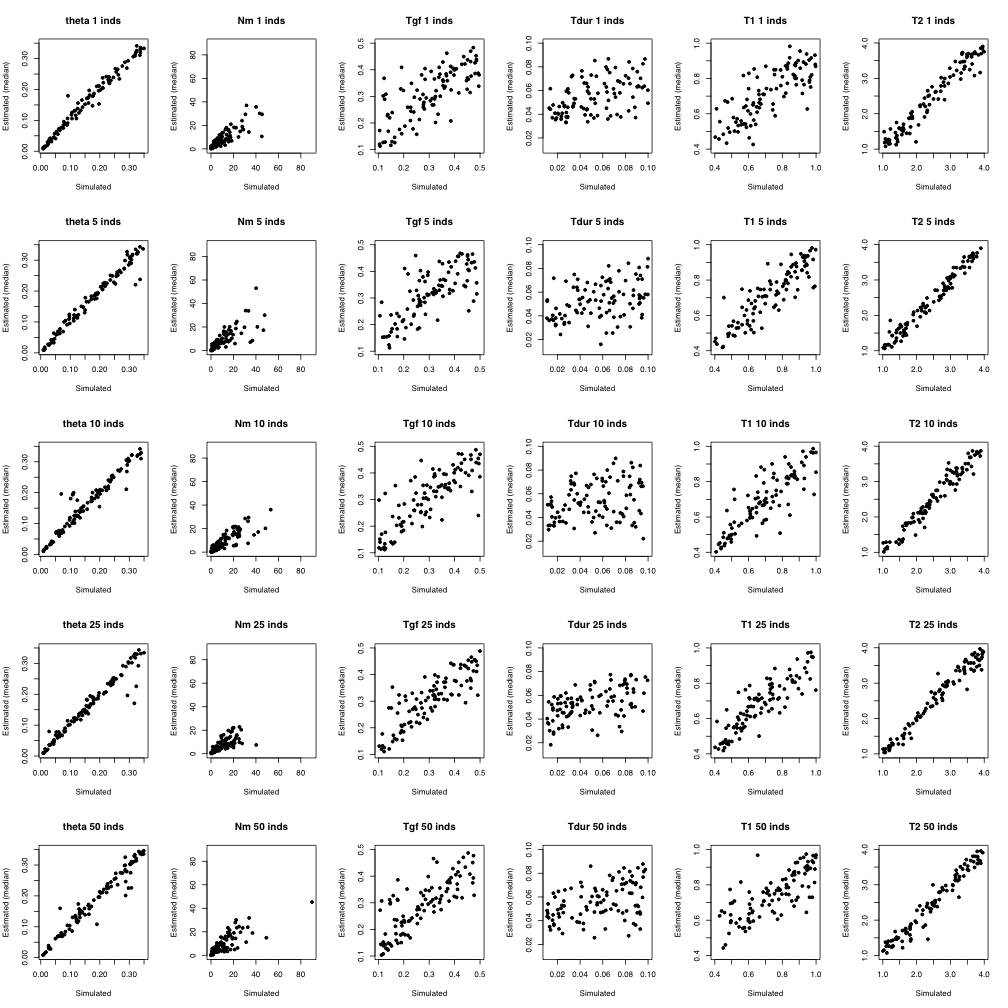


**Supporting Figure 5.**

Parameter estimates for the parameters of model ISO (columns), plotted for datasets with different numbers of sampled individuals (rows).


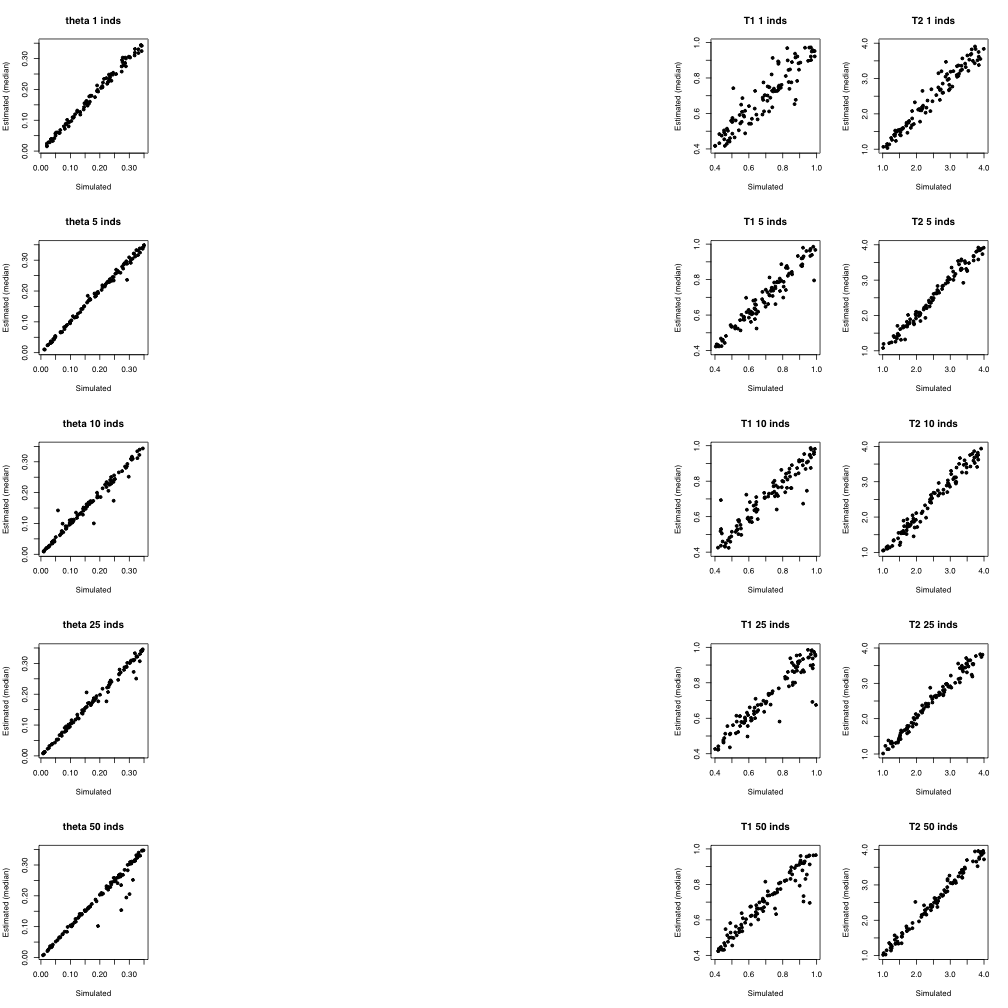


**Supporting Figure 6.**

Parameter estimates for the parameters of model A (columns), plotted for datasets with different numbers of loci (rows).


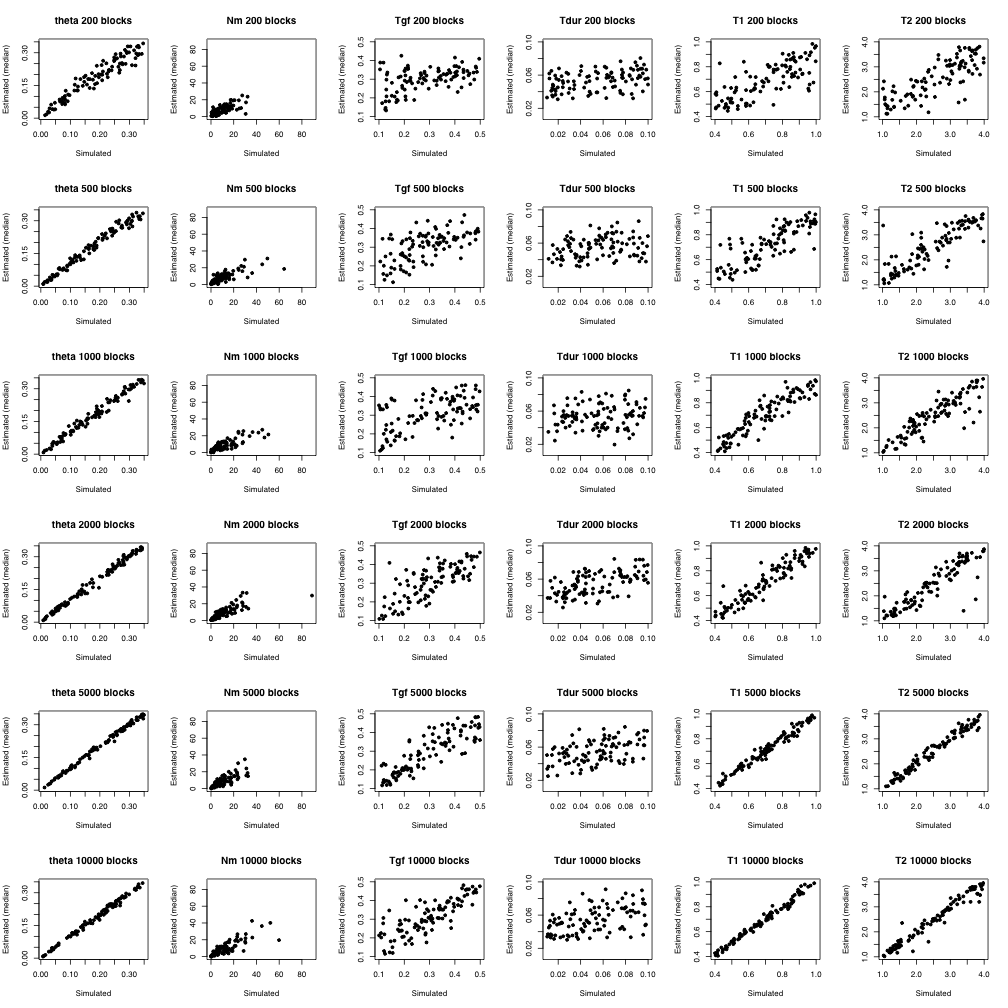


**Supporting Figure 7.**

Parameter estimates for the parameters of model C (columns), plotted for datasets with different numbers of loci (rows).


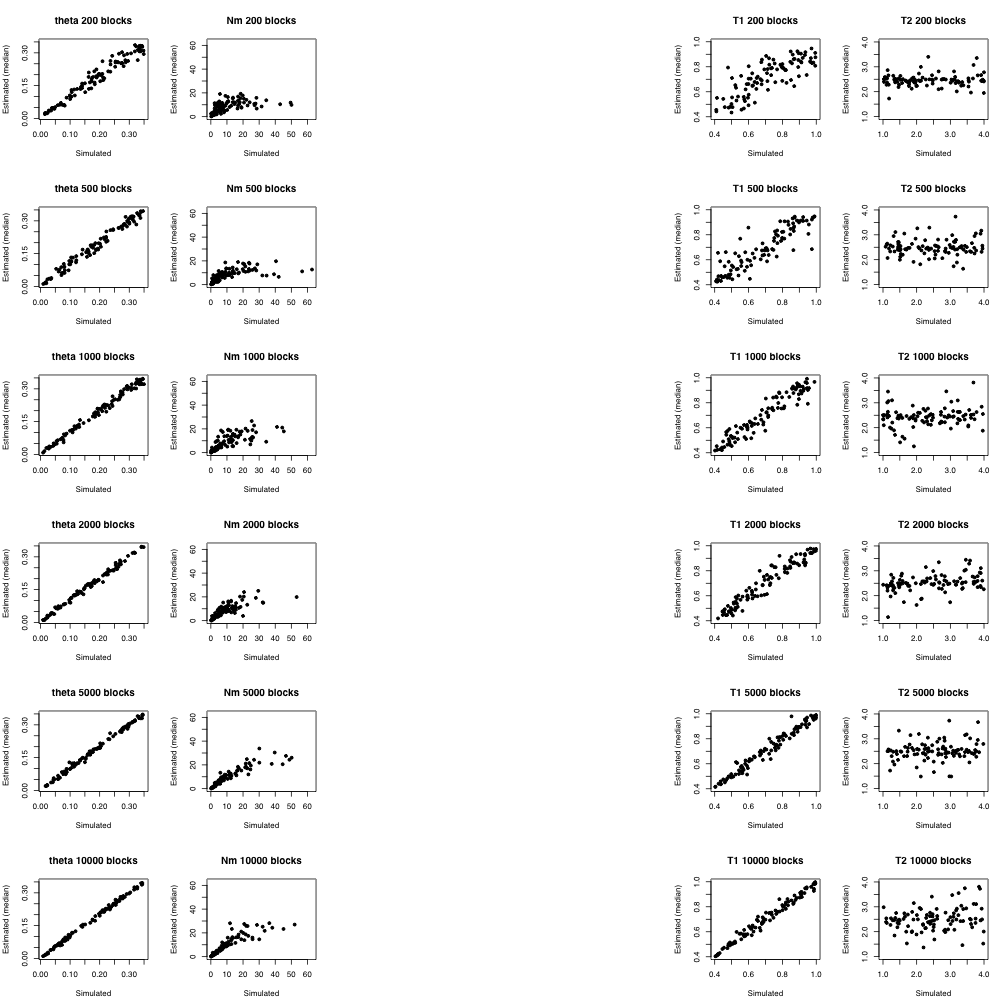


**Supporting Figure 8.**

Parameter estimates for the parameters of model D (columns), plotted for datasets with different numbers of loci (rows).


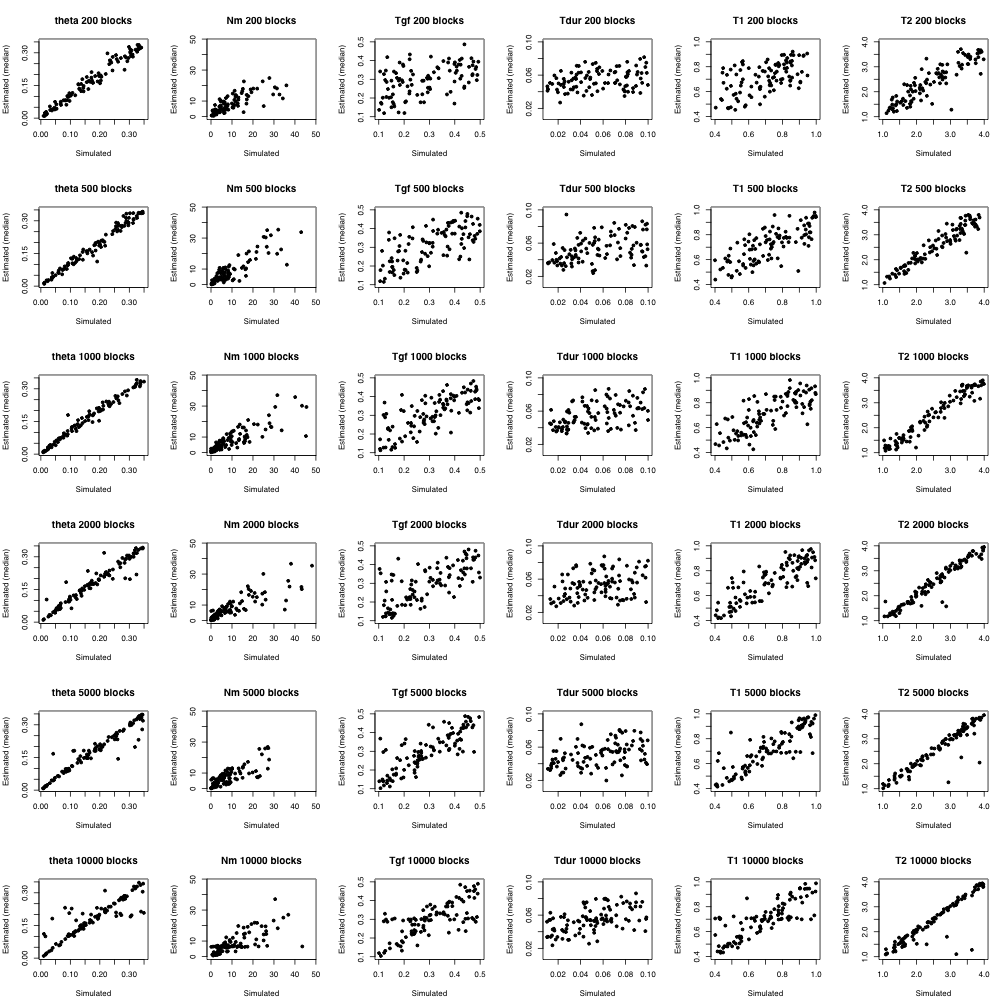


**Supporting Figure 9.**

Parameter estimates for the parameters of model ISO (columns), plotted for datasets with different numbers of loci (rows).


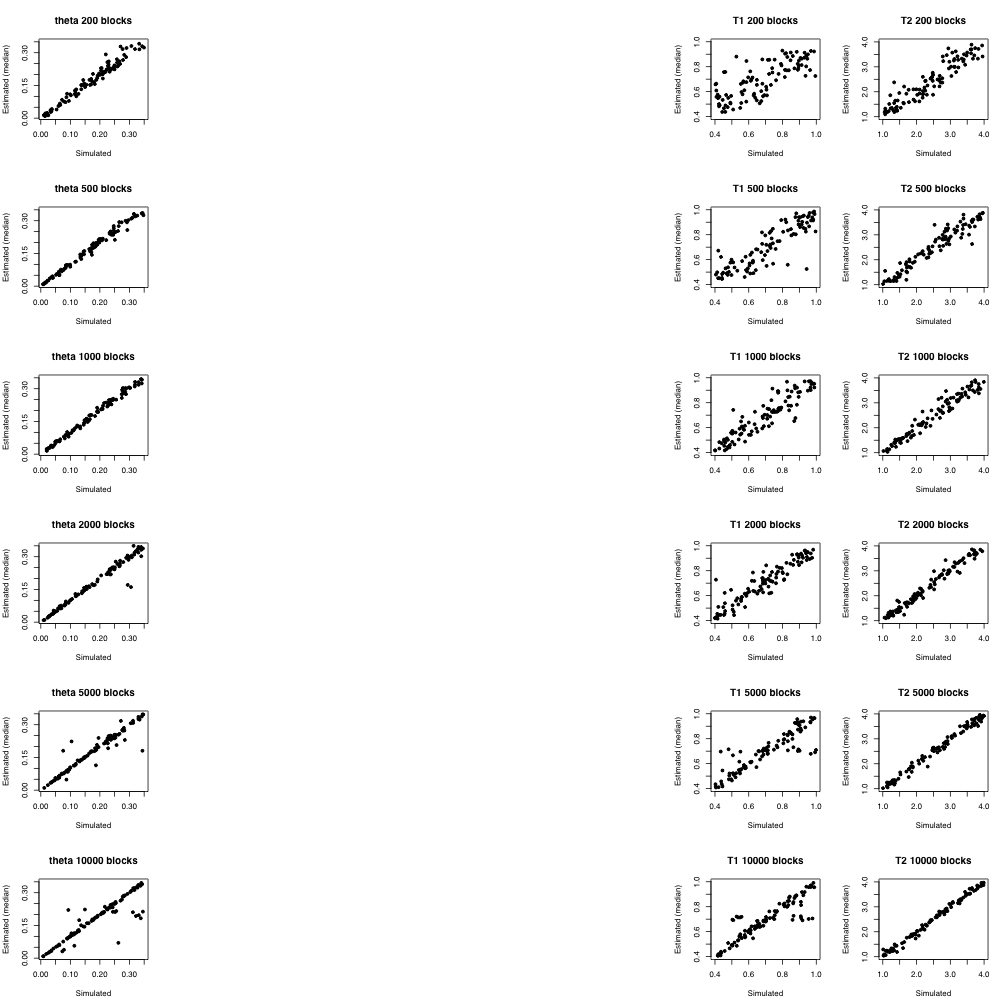


**Supporting Figure 10.**

Parameter estimates for the parameters of model A (columns), plotted for datasets with different lengths of loci (rows).


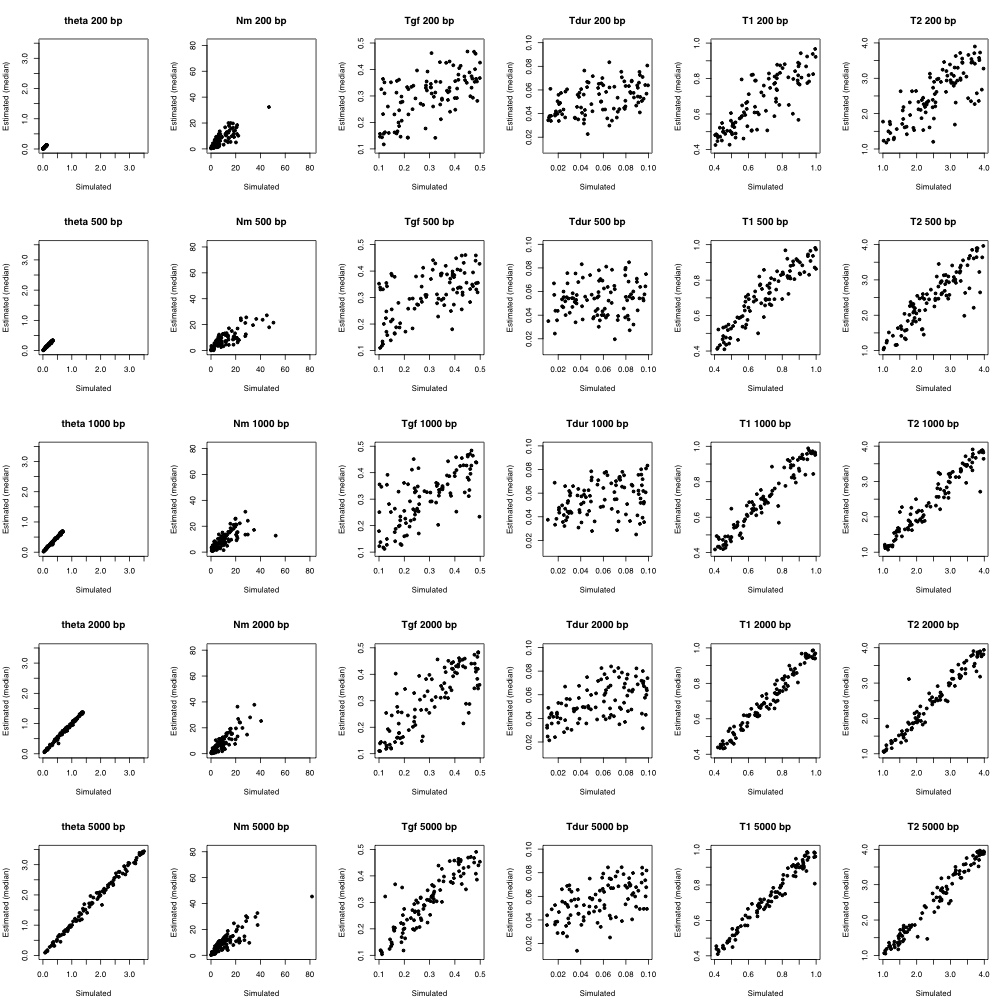


**Supporting Figure 11.**

Parameter estimates for the parameters of model C (columns), plotted for datasets with different lengths of loci (rows).


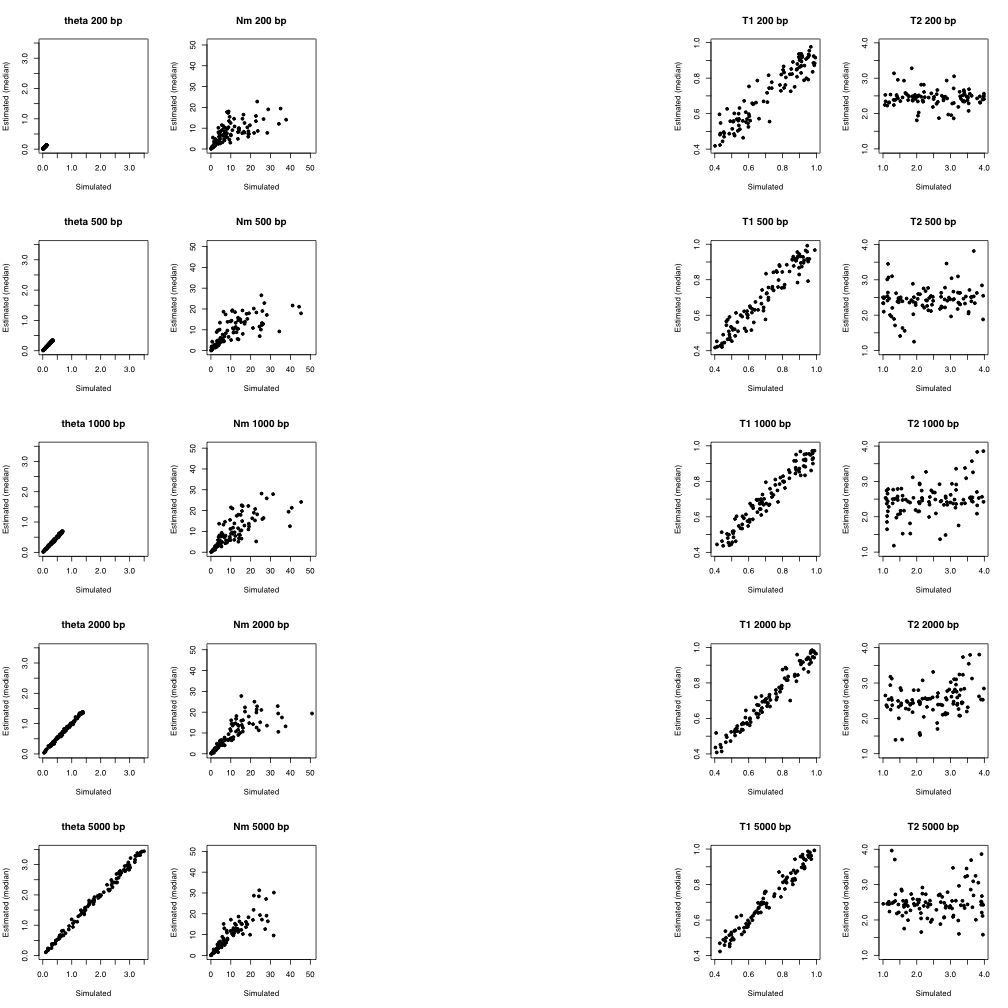


**Supporting Figure 12.**

Parameter estimates for the parameters of model D (columns), plotted for datasets with different lengths of loci (rows).


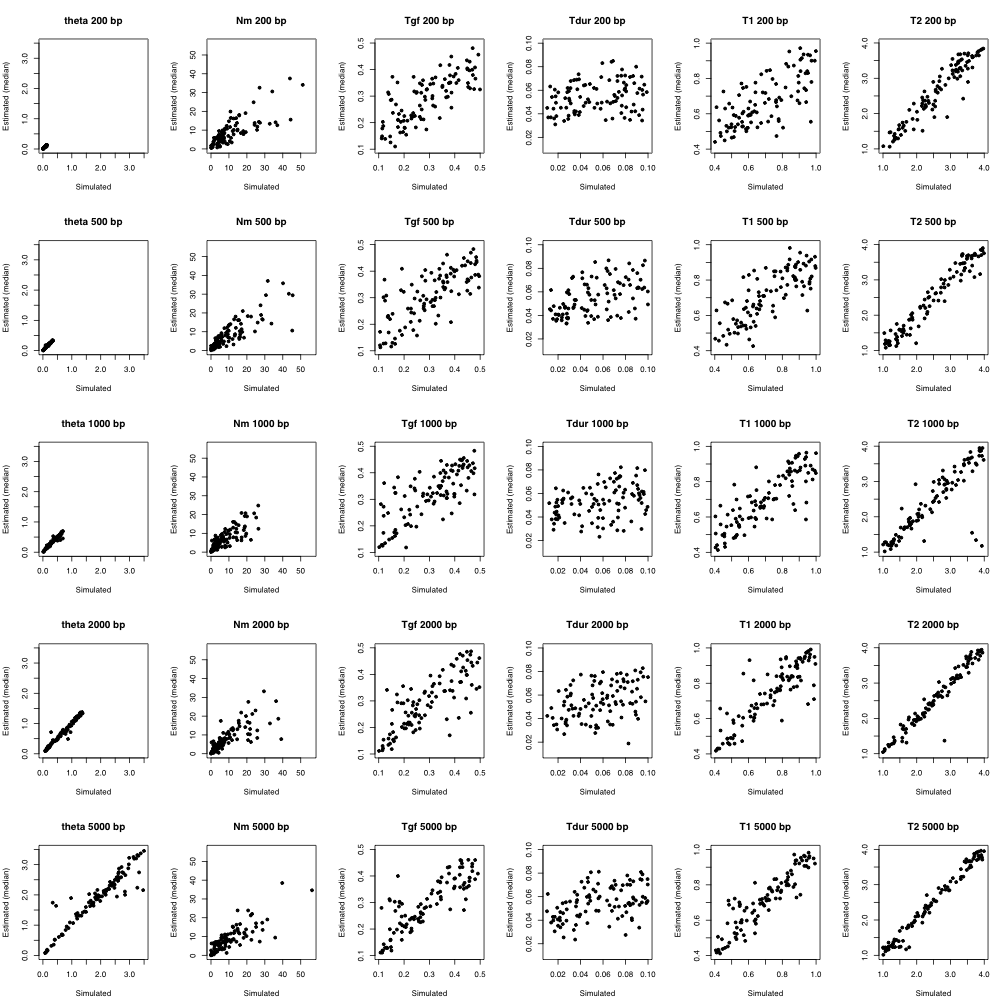


**Supporting Figure 13.**

Parameter estimates for the parameters of model ISO (columns), plotted for datasets with different lengths of loci (rows).


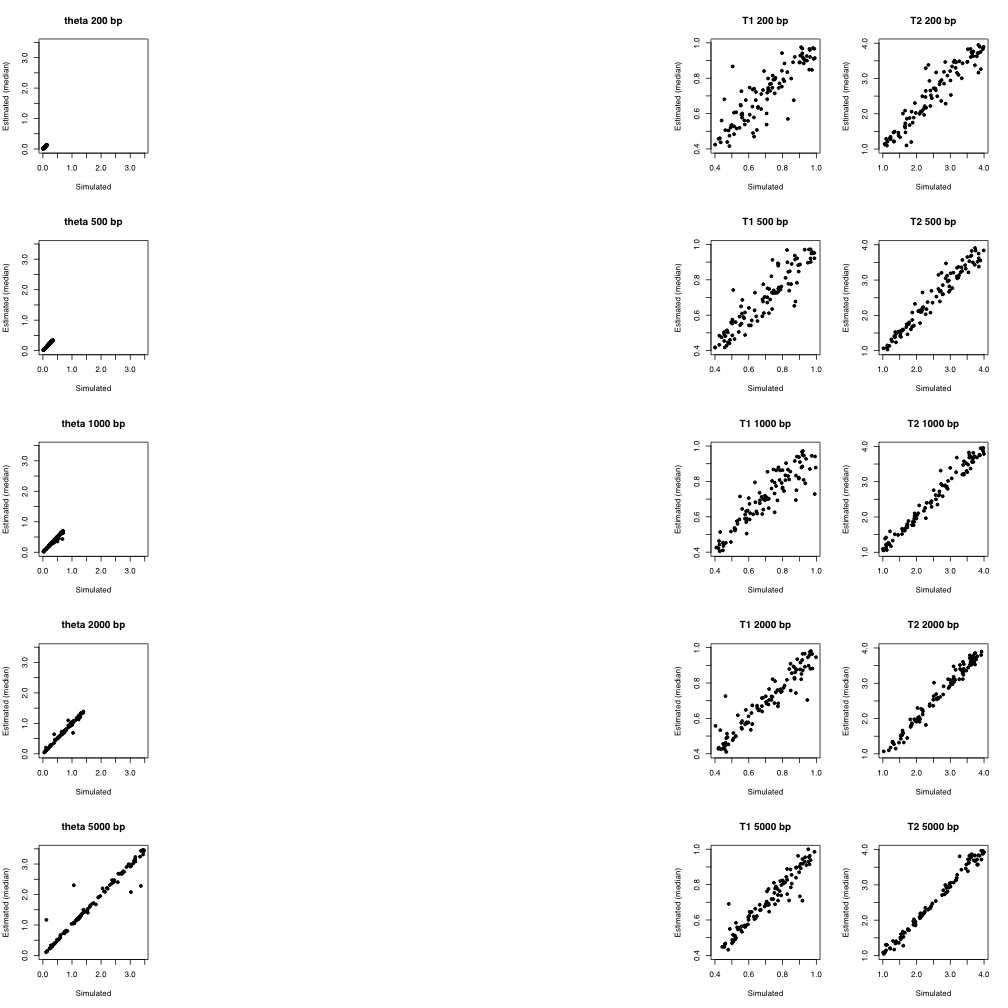


**Supporting Figures 14-16.**

Boxplots of the widths of the 95% HPD intervals for the parameters of our models (expressed as a proportion of the prior width). Models are arranged in rows, and parameters in columns. Each plot shows the influence of varying a particular sampling aspect on the width of credible intervals. Plots are in the following order: number of individuals, number of loci, length of loci.

**Supporting Figure 14.**

Widths of confidence intervals for parameters (columns) of models A, C, D, and ISO (rows), plotted separately for datasets with different numbers of sampled individuals.


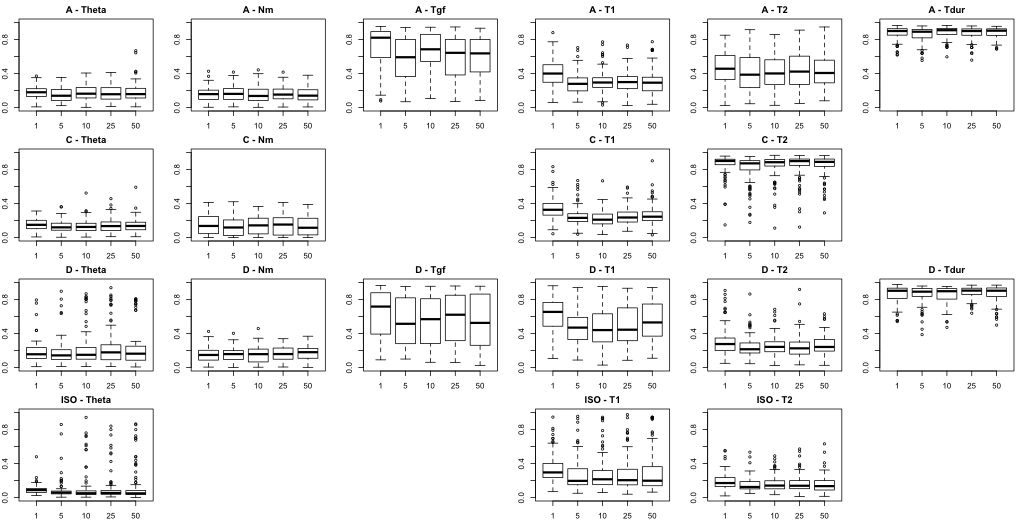


**Supporting Figure 15.**

Widths of confidence intervals for parameters (columns) of models A, C, D, and ISO (rows), plotted separately for datasets with different numbers of loci.


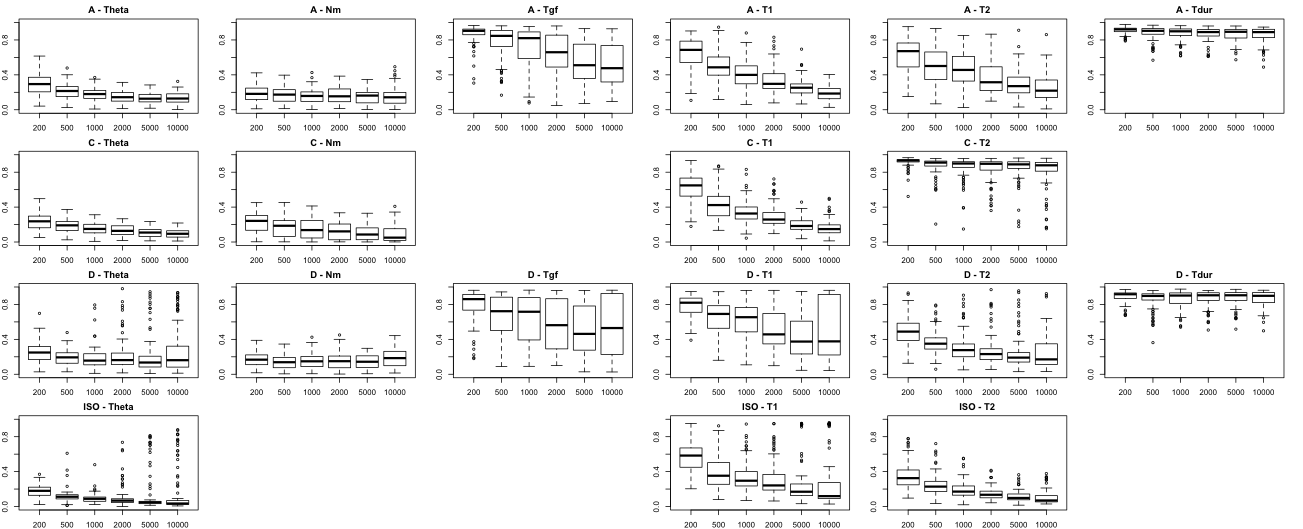


**Supporting Figure 16.**

Widths of confidence intervals for parameters (columns) of models A, C, D, and ISO (rows), plotted separately for datasets with different sizes of loci.


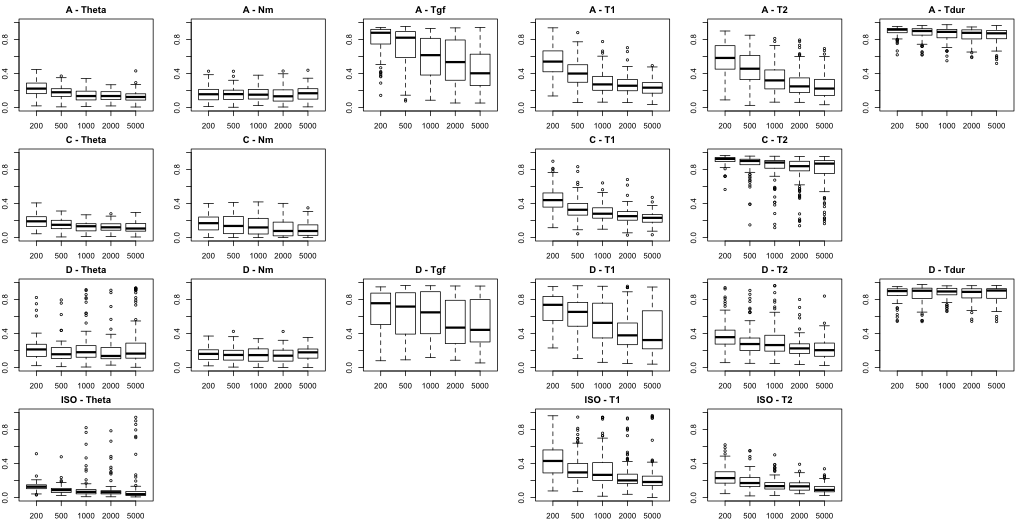


**Supporting Figure 17.**

Results for model selection analyses for simulated datasets using all 52 summary statistics (lines) and for two sampling schemes when using only the means of the statistic distributions (“X”). Plots show the mean posterior probability of the true model as the number of sequenced loci increases. The three panels in the right-hand column show the number of replicates (out of 100) where the minimum pairwise Bayes Factor in favor of the true model is greater than 10.

**
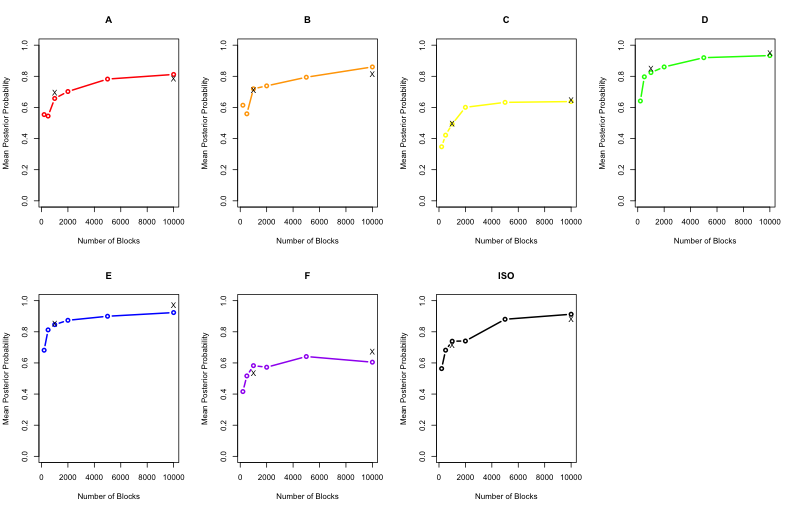
**

**Supporting Figure 18.**

Results for model selection analyses for simulated datasets using all 52 summary statistics (lines) and for two sampling schemes when using only the means of the statistic distributions (“X”). Plots show the show the number of replicates (out of 100) where the minimum pairwise Bayes Factor in favor of the true model is greater than 10.

**
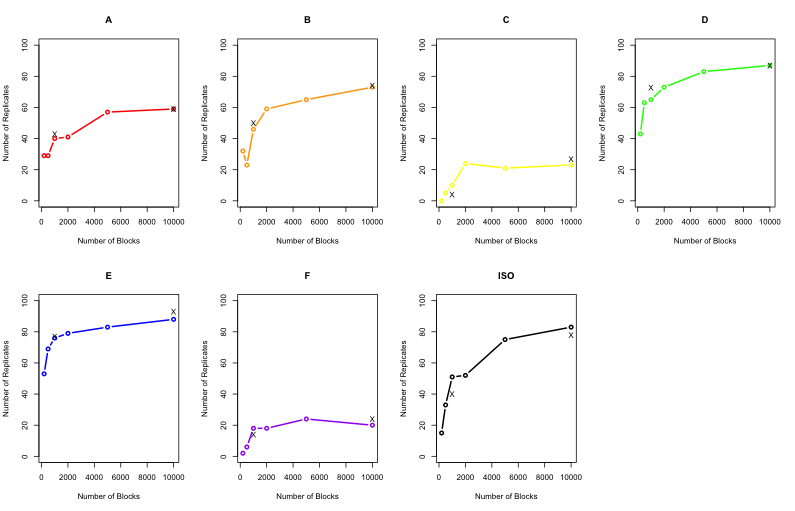
**

**Supporting Figure 19.**

Prediction errors for parameters of model A, across datasets sampling different numbers of loci using all 52 summary statistics (lines), or only the means of the statistic distributions (“X”).


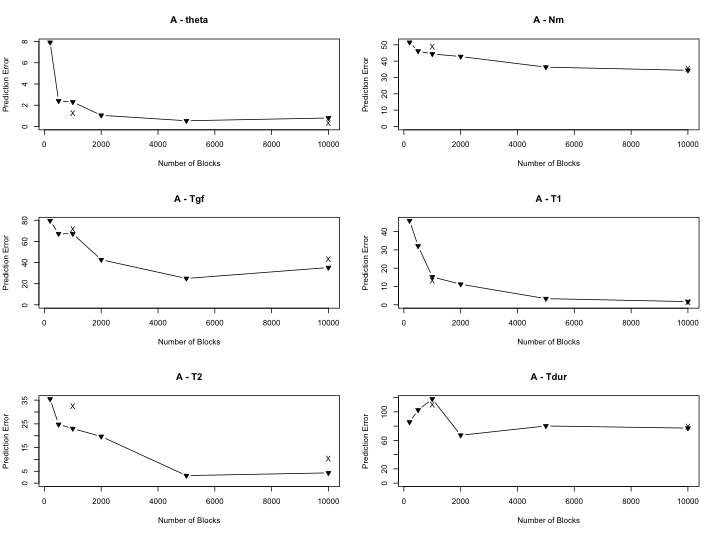


**Supporting Figure 20.**

Prediction errors for parameters of model C, across datasets sampling different numbers of loci using all 52 summary statistics (lines), or only the means of the statistic distributions (“X”).


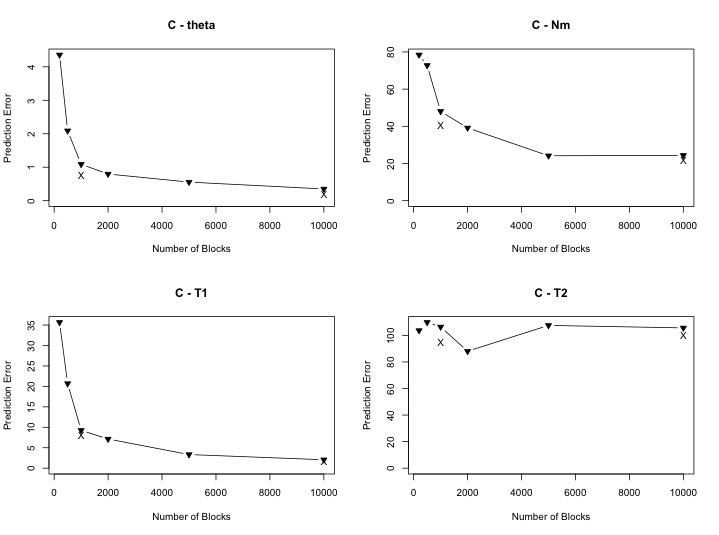


**Supporting Figure 21.**

Prediction errors for parameters of model D, across datasets sampling different numbers of loci using all 52 summary statistics (lines), or only the means of the statistic distributions (“X”).


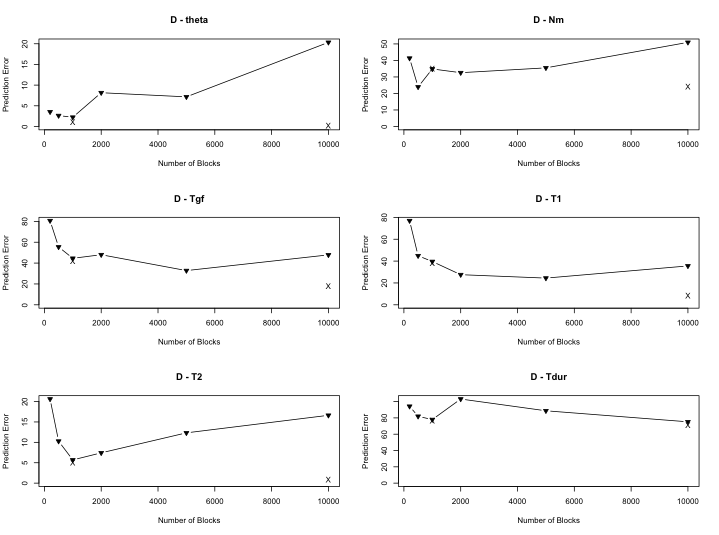


**Supporting Figure 22.**

Prediction errors for parameters of model ISO, across datasets sampling different numbers of loci using all 52 summary statistics (lines), or only the means of the statistic distributions (“X”).


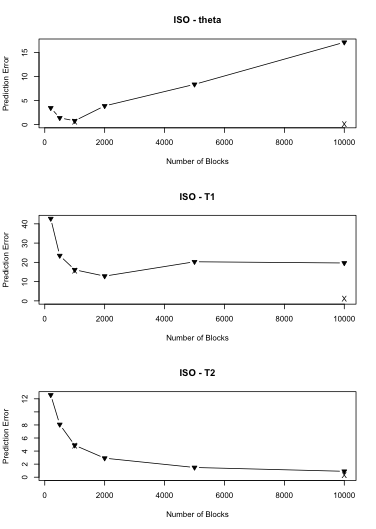
**Supporting Figure 23.**

Principal components analyses of the simulated and observed summary statistics for the seven candidate models (A-F, ISO) and the B. pallida dataset. A PCA using posterior predictive simulations, simulations with parameter value and model combinations from accepted datasets, is also plotted. The grey circle in each plot gives the location of the observed data on the first two principal components.


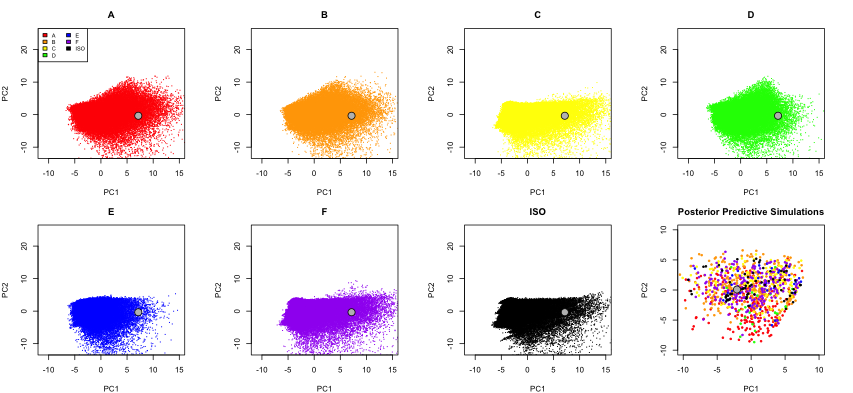

Supplement: Table S1 — Sampling strategies considered during our simulation study. Table S2 Counts of the number of single nucleotide polymorphisms (SNPs) that support different groups in the empirical dataset (1080 loci). Table S3 Observed values of the 40 summary statistics used for the ABC application to data from Biorhiza pallida. Table S4 Coverage of the 95% HPD intervals estimated for pseudo-observed datasets (PODS). Table S5 Mean posterior probabilities, and the number of replicates with strong support (minimum Bayes Factor > 10), for the simulated model under the “optimal” sampling strategy. Table S6 Prediction errors for model parameters under the “optimal” sampling strategy vs. the largest datasets simulated varying the number of individuals, the number of loci, or the lengths of loci. [file mec0023-4458-SD1.docx]
