# Supplementary material for: ABC inference of multi-population divergence with admixture from unphased population genomic data
Source: Mol Ecol. 2014 Sep 6;23(18):4458–71. doi: 10.1111/mec.12881 (PMC4285295; doi:10.1111/mec.12881)
Supplement: Fig S1 — Sampling locations for the Biorhiza pallida individuals included in our empirical application. Fig. S2 Parameter estimates for the parameters of model A (columns), plotted for datasets with different numbers of sampled individuals (rows). Fig. S3 Parameter estimates for the parameters of model C (columns), plotted for datasets with different numbers of sampled individuals (rows). Fig. S4 Parameter estimates for the parameters of model D (columns), plotted for datasets with different numbers of sampled individuals (rows). Fig. S5 Parameter estimates for the parameters of model ISO (columns), plotted for datasets with different numbers of sampled individuals (rows). Fig. S6 Parameter estimates for the parameters of model A (columns), plotted for datasets with different numbers of loci (rows). Fig. S7 Parameter estimates for the parameters of model C (columns), plotted for datasets with different numbers of loci (rows). Fig. S8 Parameter estimates for the parameters of model D (columns), plotted for datasets with different numbers of loci (rows). Fig. S9 Parameter estimates for the parameters of model ISO (columns), plotted for datasets with different numbers of loci (rows). Fig. S10 Parameter estimates for the parameters of model A (columns), plotted for datasets with different lengths of loci (rows). Fig. S11 Parameter estimates for the parameters of model C (columns), plotted for datasets with different lengths of loci (rows). Fig. S12 Parameter estimates for the parameters of model D (columns), plotted for datasets with different lengths of loci (rows). Fig. S13 Parameter estimates for the parameters of model ISO (columns), plotted for datasets with different lengths of loci (rows). Fig. S14 Widths of confidence intervals for parameters (columns) of models A, C, D, and ISO (rows), plotted separately for datasets with different numbers of sampled individuals. Fig. S15 Widths of confidence intervals for parameters (columns) of models A, C, D, and ISO (rows), plotted [file mec0023-4458-SD2.pdf]

Supporting Information – Text

Observed and Expected Prediction Error (ε)

Observed prediction errors for each parameter were calculated as,

$$\varepsilon = \frac{\sum(\hat{\theta} - \theta^*)^2}{Var(\theta^*)}$$

where $\hat{\theta}$ is the estimated parameter value (median of the posterior distribution) and θ^* is the randomly-drawn simulated value. Additionally, we calculated the expected prediction error for each parameter based on its prior distribution - that which would result if no information were available for estimation of a parameter, for which the posterior distribution would match the prior distribution. In this case, the point estimate above would correspond to the median of the prior distribution. In that case, the numerator of Eq. 1 is the summed squared deviance between the median of the prior distribution and individual values drawn from that distribution, which is very similar to the variance in the denominator of Eq. 1. Thus, for parameters with flat, uninformative prior distributions, the expected prediction error for an ABC estimator is approximately equal to the number of replicates simulated.

Computational load

One issue for ABC is the time taken to simulate model datasets for comparison with observed data. Across the seven models compared in *B. pallida*, our simulations required an average of 0.2 seconds per replicate (on a 2.6 GHz Intel i7 processor). Simulation of the entire reference table (14 million entries) would require approximately 778 hours. However, simulations can be conducted in parallel on multiple computers for ABC, and then combined before the final analysis, substantially shortening the

simulation step. For example, given access to 20 nodes on a high-performance computing cluster, the simulation portion of our empirical analysis could be completed in under two days. By comparison, Hearn et al. (2014) compared the same 7 models considered using the likelihood method of Lohse et al. (2011) in approximately 43 minutes, and all 21 models (testing all three possible topologies) required approximately 129 minutes. The likelihood method thus requires substantially less computing time for datasets of a similar size.
